# Harnessing cognitive trajectory clusterings to examine subclinical decline risk factors

**DOI:** 10.1093/braincomms/fcad333

**Published:** 2023-12-03

**Authors:** Lianlian Du, Bruce P Hermann, Erin M Jonaitis, Karly Alex Cody, Leonardo Rivera-Rivera, Howard Rowley, Aaron Field, Laura Eisenmenger, Bradley T Christian, Tobey J Betthauser, Bret Larget, Rick Chappell, Shorena Janelidze, Oskar Hansson, Sterling C Johnson, Rebecca Langhough

**Affiliations:** Wisconsin Alzheimer’s Institute, University of Wisconsin-Madison School of Medicine and Public Health, Madison, WI 53792, USA; Wisconsin Alzheimer’s Disease Research Center, University of Wisconsin-Madison School of Medicine and Public Health, Madison, WI 53792, USA; Department of Medicine, University of Wisconsin-Madison School of Medicine and Public Health, Madison, WI 53792, USA; Wisconsin Alzheimer’s Institute, University of Wisconsin-Madison School of Medicine and Public Health, Madison, WI 53792, USA; Department of Neurology, University of Wisconsin-Madison School of Medicine and Public Health, Madison, WI 53705, USA; Wisconsin Alzheimer’s Institute, University of Wisconsin-Madison School of Medicine and Public Health, Madison, WI 53792, USA; Wisconsin Alzheimer’s Disease Research Center, University of Wisconsin-Madison School of Medicine and Public Health, Madison, WI 53792, USA; Department of Medicine, University of Wisconsin-Madison School of Medicine and Public Health, Madison, WI 53792, USA; Wisconsin Alzheimer’s Institute, University of Wisconsin-Madison School of Medicine and Public Health, Madison, WI 53792, USA; Wisconsin Alzheimer’s Disease Research Center, University of Wisconsin-Madison School of Medicine and Public Health, Madison, WI 53792, USA; Department of Medicine, University of Wisconsin-Madison School of Medicine and Public Health, Madison, WI 53792, USA; Wisconsin Alzheimer’s Disease Research Center, University of Wisconsin-Madison School of Medicine and Public Health, Madison, WI 53792, USA; Department of Medical Physics, University of Wisconsin-Madison School of Medicine and Public Health, Madison, WI 53705, USA; Department of Radiology, University of Wisconsin-Madison School of Medicine and Public Health, Madison, WI 53792, USA; Department of Radiology, University of Wisconsin-Madison School of Medicine and Public Health, Madison, WI 53792, USA; Department of Radiology, University of Wisconsin-Madison School of Medicine and Public Health, Madison, WI 53792, USA; Wisconsin Alzheimer’s Disease Research Center, University of Wisconsin-Madison School of Medicine and Public Health, Madison, WI 53792, USA; Department of Medical Physics, University of Wisconsin-Madison School of Medicine and Public Health, Madison, WI 53705, USA; Waisman Laboratory for Brain Imaging and Behavior, University of Wisconsin-Madison, Madison, WI 53705, USA; Wisconsin Alzheimer’s Institute, University of Wisconsin-Madison School of Medicine and Public Health, Madison, WI 53792, USA; Wisconsin Alzheimer’s Disease Research Center, University of Wisconsin-Madison School of Medicine and Public Health, Madison, WI 53792, USA; Department of Medicine, University of Wisconsin-Madison School of Medicine and Public Health, Madison, WI 53792, USA; Department of Statistics, University of Wisconsin-Madison, Madison, WI 53706, USA; Wisconsin Alzheimer’s Disease Research Center, University of Wisconsin-Madison School of Medicine and Public Health, Madison, WI 53792, USA; Department of Biostatistics and Medical Informatics, School of Medicine and Public Health, University of Wisconsin-Madison, Madison, WI 53726, USA; Clinical Memory Research Unit, Lund University, Lund 205 02, Sweden; Clinical Memory Research Unit, Lund University, Lund 205 02, Sweden; Wisconsin Alzheimer’s Institute, University of Wisconsin-Madison School of Medicine and Public Health, Madison, WI 53792, USA; Wisconsin Alzheimer’s Disease Research Center, University of Wisconsin-Madison School of Medicine and Public Health, Madison, WI 53792, USA; Department of Medicine, University of Wisconsin-Madison School of Medicine and Public Health, Madison, WI 53792, USA; Wisconsin Alzheimer’s Institute, University of Wisconsin-Madison School of Medicine and Public Health, Madison, WI 53792, USA; Wisconsin Alzheimer’s Disease Research Center, University of Wisconsin-Madison School of Medicine and Public Health, Madison, WI 53792, USA; Department of Medicine, University of Wisconsin-Madison School of Medicine and Public Health, Madison, WI 53792, USA

**Keywords:** preclinical trajectories, neurodegeneration, amyloid, vascular, change point

## Abstract

Cognitive decline in Alzheimer’s disease and other dementias typically begins long before clinical impairment. Identifying people experiencing subclinical decline may facilitate earlier intervention. This study developed cognitive trajectory clusters using longitudinally based random slope and change point parameter estimates from a Preclinical Alzheimer’s disease Cognitive Composite and examined how baseline and most recently available clinical/health-related characteristics, cognitive statuses and biomarkers for Alzheimer’s disease and vascular disease varied across these cognitive clusters. Data were drawn from the Wisconsin Registry for Alzheimer’s Prevention, a longitudinal cohort study of adults from late midlife, enriched for a parental history of Alzheimer’s disease and without dementia at baseline. Participants who were cognitively unimpaired at the baseline visit with ≥3 cognitive visits were included in trajectory modelling (*n* = 1068). The following biomarker data were available for subsets: positron emission tomography amyloid (amyloid: *n* = 367; [^11^C]Pittsburgh compound B (PiB): global PiB distribution volume ratio); positron emission tomography tau (tau: *n* = 321; [^18^F]MK-6240: primary regions of interest meta-temporal composite); MRI neurodegeneration (neurodegeneration: *n* = 581; hippocampal volume and global brain atrophy); T_2_ fluid-attenuated inversion recovery MRI white matter ischaemic lesion volumes (vascular: white matter hyperintensities; *n* = 419); and plasma pTau217 (*n* = 165). Posterior median estimate person-level change points, slopes’ pre- and post-change point and estimated outcome (intercepts) at change point for cognitive composite were extracted from Bayesian Bent-Line Regression modelling and used to characterize cognitive trajectory groups (*K*-means clustering). A common method was used to identify amyloid/tau/neurodegeneration/vascular biomarker thresholds. We compared demographics, last visit cognitive status, health-related factors and amyloid/tau/neurodegeneration/vascular biomarkers across the cognitive groups using ANOVA, Kruskal–Wallis, *χ*^2^, and Fisher’s exact tests. Mean (standard deviation) baseline and last cognitive assessment ages were 58.4 (6.4) and 66.6 (6.6) years, respectively. Cluster analysis identified three cognitive trajectory groups representing steep, *n* = 77 (7.2%); intermediate, *n* = 446 (41.8%); and minimal, *n* = 545 (51.0%) cognitive decline. The steep decline group was older, had more females, *APOE* e4 carriers and mild cognitive impairment/dementia at last visit; it also showed worse self-reported general health-related and vascular risk factors and higher amyloid, tau, neurodegeneration and white matter hyperintensity positive proportions at last visit. Subtle cognitive decline was consistently evident in the steep decline group and was associated with generally worse health. In addition, cognitive trajectory groups differed on aetiology-informative biomarkers and risk factors, suggesting an intimate link between preclinical cognitive patterns and amyloid/tau/neurodegeneration/vascular biomarker differences in late middle-aged adults. The result explains some of the heterogeneity in cognitive performance within cognitively unimpaired late middle-aged adults.

## Teaser

Du *et al*. identified cognitive trajectory profiles using person-level slopes and change point from a composite in late middle-aged adults. The groups were associated with differences in health/lifestyle and dementia-related (A/T/N/V) biomarkers. Identifying person-specific subclinical decline may provide an opportunity for early intervention.

## Graphical Abstract

**Graphical Abstract:**
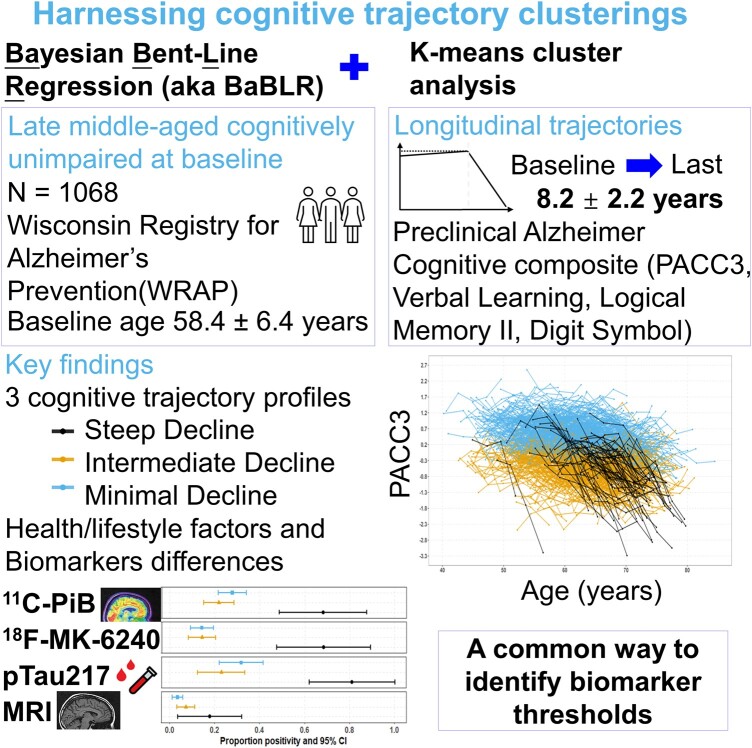

## Introduction

Individuals who ultimately receive a diagnosis of mild cognitive impairment (MCI) or dementia typically have observable cognitive changes many years prior to diagnosis.^1,2^ These preclinical cognitive trajectories are heterogeneous,^3,4^ and the processes that impact differential rates of cognitive change within the preclinical phase remain only partially understood. For example, studies including the cohort used in this paper have shown that the presence and duration/amount of amyloid and tau proteinopathies are associated with worse cognitive trajectories prior to any diagnosis of MCI or dementia due to Alzheimer’s disease.^5–7^ This was highly consistent with the amyloid/tau/neurodegeneration (ATN) framework for characterizing the biological state of Alzheimer’s disease, independent of clinical manifestation.^8^ Cerebrovascular ischaemic disease has also been associated with cognitive decline and increased risk of dementia.^9^ White matter hyperintensities (WMH) in this context are thought to reflect tissue damage from vascular (V) ischaemia and have been associated with poor cognitive outcomes.^10^ Other studies^11,12^ have examined health factors and medical conditions in relationship to cognitive trajectories. For example, Junhong *et al*.^11^ observed that lower medical burden (as operationalized by the absence of hypertension, diabetes and heart diseases) and healthier lifestyles (e.g. do not smoke or drink and exercise regularly) at baseline were associated with the most resilient cognitive trajectories in the late-life period. In other studies, self-reported memory problems and *APOE* e4 status have been shown to be associated with faster preclinical cognitive decline^13,14^ or increased risk of progression to dementia.^15,16^ Thus, a wide and diverse range of risk factors for early cognitive decline have been identified, subsets of which have been examined across a diversity of studies.

There are several defining features associated with progression to eventual dementia, including an accelerated decline in cognitive function that tends to occur after a certain time point [or ‘change point’ (CP)]. Change point analysis methods constitute a valuable framework for the investigation of the timing of cognitive decline and the rate of changes.^17^ They are a simplification in that change may not be resolvable to a single point but may nevertheless approximate a heterogeneous time course at the person level. A longitudinal evaluation of the preclinical period has offered insight into when older adults undergo a CP over the course of cognitive aging, indicating an acceleration in the rate of cognitive decline preceding diagnosis (i.e. MCI or dementia) or death.^17^ These CPs often appear years before diagnosis or death and vary across cognitive domains, providing insight into the sensitivity of different indicators that exhibit the earliest onset of accelerated decline. MCI and Alzheimer’s disease patients evince faster cognitive decline compared to healthy controls for a range of cognitive domains including memory, executive function, attention and verbal fluency, but these steeper rates of decline ranged broadly from 1.9 to 15.5 years across cognitive measures prior to Alzheimer’s disease diagnosis.^4^ Change point models have been used to study the temporal relationship of various biomarkers as well, leading to models that are partially consistent with the theoretical model.^18^ Du *et al*.^19^ recently developed Bayesian Bent-Line Regression (aka BaBLR) modelling to estimate within-individual transition points, presumably signalling a transition from normal, healthy aging to abnormal cognitive change. Using this method, we could estimate fixed (group-level) and random (person-level) CPs, slopes’ pre- and post-CP and age intercepts at CP for longitudinal data. The heterogeneous trajectories may be harnessed with cluster analysis and examined relative to dementia risk factors.^20,21^

The within-person preclinical cognitive trajectory patterns are not fully understood. To address this knowledge gap, this study examined the complex array of aforementioned dementia risk factors simultaneously in a baseline cognitively unimpaired sample with well-characterized within-person preclinical cognitive trajectory patterns using data from the Wisconsin Registry for Alzheimer’s Prevention (WRAP). The primary aims of this study were as follows: (i) to use *K*-means clustering with BaBLR-derived random effects from a cognitive composite patterned after Donohue *et al*.^22^ and described by Jonaitis *et al*.^23^ to identify cognitive trajectory subgroups and (ii) to examine how these subgroups differed in terms of a comprehensive array of demographic characteristics, health-related factors and A, T, N and V biomarkers.

## Materials and methods

### Participants

The WRAP study includes data from participants who enrolled at midlife and were free of dementia at baseline [mean_age_ (SD) = 54.4 (6.7) years; 94.3% between ∼40 and 65 years of age; 70.7% female; 73.3% with a parental family history of Alzheimer’s disease; ∼70% retention; see Johnson *et al.*^24^]. The study, which began in 2001, is designed to identify early cognitive decline and to characterize midlife factors (e.g. Alzheimer’s disease biomarkers and health-related factors) associated with such decline. All neuropsychological follow-up visits currently occur at ∼2-year intervals. The study is conducted in compliance with ethical principles for human subjects research defined in the Declaration of Helsinki, including review and approval by the University of Wisconsin Institutional Review Board and the provision of informed consent by all participants. Of the 1609 enrolled at the time of these analyses, individuals were excluded from BaBLR analyses if they had fewer than three cognitive visits containing the tests needed to derive a three-test preclinical alzheimer’s cognitive composite (PACC3) scores (*n* = 541). All individuals were non-demented at the time of their first PACC3. Biomarker samples were included for WRAP participants who had at least one corresponding biomarker measurement, resulting in different sample sizes for positron emission tomography (PET) amyloid (A: *n* = 367); PET tau (T: *n* = 321); MRI neurodegeneration (N: *n* = 581); T_2_ fluid-attenuated inversion recovery (FLAIR) MRI white matter ischaemic lesion volumes (V: *n* = 419); and plasma pTau217 (*n* = 165).

### Clinical and cognitive assessments

At each study visit, WRAP participants completed a comprehensive cognitive battery described in full elsewhere.^24^ We assessed longitudinal cognitive performance using a three-test version of the modified Preclinical Alzheimer’s Cognitive Composite (PACC) score^23^ that included the Rey Auditory Verbal Learning Test (AVLT; Trials 1–5),^25^ the Wechsler Memory Scale Logical Memory II^26^ and the Wechsler Adult Intelligence Scale-Revised Digit Symbol Substitution.^27^ The Logical Memory II was added when Visit 2 was initiated; thus, PACC3 baseline is Visit 2 for most participants (94%) and Visit 1 for recent enrolees. Each contributing test score was rescaled to a *Z*-score using the mean and standard deviation of the baseline score among cognitively unimpaired individuals. For use in the BaBLR model (described in the analysis section), the unweighted average of these *Z*-scores was then converted to the PACC3 *Z*-score using the mean and standard deviation of the baseline PACC3 score among cognitively unimpaired individuals.

#### Self-reported memory

Subjective complaint measures included two items representing participants’ self-report of memory functioning: ‘Do you think you have a problem with your memory?’ (0 = no, 1 = yes; ‘Don’t know’ coded to missing) and ‘Overall, how would you rate your memory in terms of the kinds of problems that you have?’ from the Memory Functioning Questionnaire.^28^ The Likert scale scores were summarized as 1–3 = major problems, 4 = neutral and 5–7 = no problems (reference group).

### Consensus-based cognitive status

Participant cognitive status at each visit was determined via multi-disciplinary consensus conference as described comprehensively elsewhere.^29^ Briefly, participants were reviewed by the multi-disciplinary consensus team after a study visit if a participant met screening criteria using internal norms,^30,31^ raw score cut-offs or informant report cut-offs. At consensus review, the team determined whether dementia or MCI was present. If these impairments were absent, some were assigned a status of ‘Cognitively Unimpaired-Declining’ (CU-D) denoting subtle cognitive impairment consistent with a trajectory towards MCI or dementia but not reaching clinical thresholds of impairment, and others were assigned a status of ‘Cognitively Unimpaired-Stable’ (CU-S).

### *APOE* genotypes

*APOE* ɛ2/ɛ3/ɛ4 and 20 common genetic variants from the International Genomics of Alzheimer’s Project consortium were genotyped using competitive allele-specific polymerase chain reaction–based genotyping assays (LGC Genomics, Beverly, MA) as described previously.^24^ For comparisons, *APOE* ɛ4 status was dichotomized as positive or negative, indicating presence or absence of any ɛ4 alleles, respectively.

### Health-related factors

#### Medication use

Medications at each visit were quantified by a combination of self-report itemization, medical records and by medications brought to the study visit. The total number of prescriptions was counted for each participant at each visit, up to 15 prescriptions. For comparisons, three groups were created: no polypharmacy (0–4 medications; reference group), polypharmacy (5–9 medications) and hyperpolypharmacy (≥10 medications).^12^

#### Comorbid disease tally

The comorbidity tally was defined at each study visit as the sum of self-reported chronic conditions (e.g. heart disease, hypertension, diabetes and liver disorder), up to 15 comorbidities, which could be of potential interest and/or confound analyses (detailed by our group previously, see Du *et al.*^12^).

#### Body mass index and waist-to-hip ratio

Body mass index (BMI) was calculated as kilograms per square metre using height (m) and weight (kg) data collected at each visit. Similarly, waist-to-hip ratio (WHR) was calculated as measured waist (centimetres) and hip circumferences (centimetres). For comparisons, BMI was categorized as underweight (<18.5; reference group), normal weight (18.5–24.9), overweight (25.0–29.9) and obese (30.0 or above). WHR was categorized as low (0.80 or lower for women, 0.95 or lower for men; low = reference group), moderate (0.81–0.85 for women, 0.96–1.0 for men) and high (0.86 or higher for women, 1.0 or higher for men).

#### Self-rated current health

Self-rated health (SRH) was measured using a 5-point scale in response to the question, ‘How would you rate your current health?’ The Likert scale ratings were summarized as 1–2 = poor, 3 = good and 4–5 = excellent (the latter was used as the reference group for analyses).

#### Depressive symptoms

The presence of depressive symptoms was indexed using the 20-item Center for Epidemiologic Studies Depression Scale (CES-D),^32^ which was completed by each participant at each visit. Depression was defined as self-reported depression or, if no self-report, CES-D score ≥16.

#### LIfestyle for BRAin health index

As detailed elsewhere,^5^ WRAP’s modified version of the LIfestyle for BRAin health (LIBRA) index incorporated 11 modifiable risk and protective factors for dementia including low/moderate alcohol consumption and the presence of cardiovascular disease, physical inactivity, renal dysfunction, diabetes, high cholesterol, smoking, obesity, hypertension, depression and high cognitive activity. The risk scores were calculated using previously established relative risks from large epidemiological studies^33^ with higher scores indicating a higher lifestyle-related risk of dementia. For comparisons, three LIBRA risk groups were created based on LIBRA tertiles [low risk: LIBRA scores between −4.2 and 0 (reference group); moderate risk: LIBRA scores between 0.1 and 2.0; high risk: LIBRA scores between 2.1 and 8.1].

### PET and MRI imaging methods

Participants underwent amyloid PET with [^11^C]Pittsburgh compound B (PiB) and tau PET with [^18^F]MK-6240 as well as 3.0 T MRI including a T_1_-weighted 3D volume and a T_2_ FLAIR 3D volume. Detailed imaging methods have been previously described.^34,35^

#### PET amyloid

‘Amyloid burden’ was assessed as a global cortical average PiB distribution volume ratio (DVR) from the dynamic 0–70 min scan using the cerebellar grey matter as the reference region. The cortical average DVR used eight bilateral regions of interest (ROIs), selected on the basis of Alzheimer’s disease sensitivity and known amyloid binding as previously reported.^36,37^ ‘Estimated amyloid onset age’ (EAOA), defined as the approximate age in years that an individual has had suprathreshold amyloid positivity, was derived by averaging the output of two amyloid duration models, one using group-based trajectory modelling (GBTM^7^) and the other using sampled iterative local approximation.^38^

#### PET tau

‘Tau burden’ was assessed as [^18^F]-MK-6240 standardized uptake volume ratio (SUVR; 70–90 min; inferior cerebellar grey reference region excluding the superior medial vermis). For ROI analyses, volume-weighted mean SUVR was calculated for composite regions representing two bilateral volume-weighted averages of the component ROI’s: medial temporal lobe (MTL) composite (early tau stage composite, based on an average of the entorhinal cortex, hippocampus and amygdala)^39^ and meta-temporal composite [MTC; mid-to-late tau stage composite, based on Mayo MTC (an average of the entorhinal cortex, amygdala, parahippocampal, fusiform, inferior temporal and middle temporal)].^40^

#### Plasma pTau217

Ethylenediaminetetraacetic acid plasma was collected at the time of each in-person cognitive visit since 2011. Samples were processed as described by Jonaitis *et al*.^41^ A subset of WRAP who had amyloid and tau PET imaging were selected for plasma pTau217, which was assayed using the Lilly immunoassay on the Meso Scale Discovery platform, and the assays were performed at Lund University as described by Jonaitis *et al.*,^41^ Palmqvist *et al.*,^42^ Ashton *et al.*^43^ and Mattsson-Carlgren *et al.*^44^ All samples were assayed blind to any clinical and imaging data.

#### MRI hippocampal volume

Hippocampal volume (HV) was estimated in cubic centimetres using the completely automated FSL-FIRST algorithm on T_1_-weighted images.^22^

#### MRI global brain atrophy

A measure of global brain atrophy (GBA) was calculated as the ratio of CSF volume to the sum of the gray matter (GM) and WM tissue volumes in litres [CSF/(GM + WM)] segmented from T_1_-weighted images using SPM12.^45^

#### MRI white matter hyperintensity volumes

The Lesion Segmentation Tool (LST) version 1.2.3 in SPM12 was used to segment ischaemic lesions (see Johnson *et al.*^24^ and Vesperman *et al.*^46^) and calculate the total volume of WMH in cubic centimetres from 3D T_2_-weighted FLAIR scans (acquired using the GE Cube sequence, which was implemented in this protocol in 2014). These ROIs were subsequently reviewed by research staff for quality assurance, and any potential imaging abnormalities were adjudicated by a neuroradiologist (L.E.).

### Biomarker positivity methods

Biomarker positivity was determined as follows for the aforementioned biomarkers. First, PiB positivity (A+) was defined as global PiB DVR >1.16 (corresponding centiloid of 17.7, previously shown to predict accumulation^38,47^). Second, we transformed each of the other biomarkers to a *Z*-scale relative to the PiB PET A-, cognitive unimpaired (CU) subset. The *Z*-scores of MK-6240 MTL and MTC, along with plasma pTau217, were calculated as shown in Eq. (1). *Z*-scores for MRI HV, GBA and white matter hyperintensity volume (WMHV) were generated using regression parameters that adjusted for total intracranial volume (TICV) as shown in Eq. (2) [where TICV(L) = GM + WM + CSF volumes from SPM12 segmentation].


(1) $$Z\text{-}\mathrm{score}\;=\left(\frac{\mathrm{Observed}\;\mathrm{value}\text{–}\mathrm{Mean}\;\mathrm{value}\;\mathrm{of}\;\mathrm{CU},\;\mathrm{PET}\;A\text{-}}{\mathrm{Standard}\;\mathrm{deviation}\;\mathrm{of}\;\mathrm{CU},\;\mathrm{PET}\;A\text{-}}\right).$$

(2) $$Z\text{-}\mathrm{score}\;=\left(\frac{\mathrm{Observed}\;\mathrm{value}\;\text{–}\mathrm{Predicted}\;\mathrm{value}}{\mathrm{Root}\;\mathrm{mean}\;\mathrm{squared}\;\mathrm{error}\;\mathrm{from}\;\mathrm{CU},A\text{-}\mathrm{regression}\;\mathrm{model}\;\mathrm{that}\;\mathrm{adjusted}\;\mathrm{for}\;\mathrm{TICV}}\right).$$

For primary analyses of biomarker positivity, we chose a 1.5 *Z*-score cut-off for biomarkers where higher values indicate pathological changes and −1.5 for biomarkers where lower values indicate abnormality. In secondary analysis, biomarker positivity was defined using Gaussian mixture models (GMM) applied to each of the biomarker *Z*-scores. GMM modelling was performed using the ‘Mclust’ package in R.^48^ Additional details are presented in the Supplementary material.

### Statistical analyses

Statistical analyses were performed in R (Version 4.0.2^49^). To assess potential selection bias associated with our sample selection for this study, we compared demographic characteristics of WRAP participants who met inclusion criteria (*n* = 1068) versus those who did not (*n* = 541) using tests appropriate to the distribution of each variable (e.g. *χ*^2^ for categorical).

#### Aim 1 analyses

In preparation for identifying subgroups of cognitive trajectories, we first applied our BaBLR modelling to longitudinal PACC3 data. Briefly, the BaBLR method uses Bayesian modelling of longitudinal data to estimate the following fixed (group-level) and random (person-level) parameters: CPs, intercepts at CPs and slopes’ pre- and post-CP (the model illustration plot is shown in Supplementary Fig. 1). For comprehensive BaBLR method details and simulation studies showing predictive validity, please see the Supplementary material and Du *et al*.^19^ We extracted the posterior median estimated person-level parameters and identified the optimal number of cognitive clusters using the ‘Nbclust’ package in R.^50^ Up to 10 clusters were considered; Caliński and Harabasz criteria^51^ indicated that three clusters were statistically optimal, and visual inspection of spaghetti plots of the groups indicated these groups represented clinically meaningful separation between groups.

#### Aim 2 analyses

The differences between resulting cognitive trajectory groups in demographic, health, cognitive status, amyloid, tau, neurodegeneration and plasma tau measures across the cognitive trajectory clusters were examined using tests appropriate for the distributions of the variables, including *χ*^2^ or Fisher’s exact test for categorical variables and ANOVA or Kruskal–Wallis for continuous variables. Significant omnibus tests (*P* < 0.05) were followed with pairwise comparisons. Benjamini–Hochberg (BH) corrections controlling false discovery rate at 5% were applied across outcomes. Since cognitive trajectory groups differed on average age and male/female proportions, we conducted sensitivity analyses of continuous outcomes, adjusting for age and sex. Magnitudes of between-group differences were characterized using Cramer’s *V* for *χ*^2^ and Fisher’s exact test or Cliff’s delta for ANOVA and Kruskal–Wallis tests. The effect size estimates were calculated using the ‘effsize’ package in R.^52^

## Results

In the 1068 participants included in the BaBLR analyses, the average age [*M*_age_ (SD)] at the first PACC3 cognitive assessment was 58.4 (6.4) years (range 40.2–73.6) with a mean interval of 8.2 (2.2) years between the first and last cognitive assessment. Additional sample demographics and baseline characteristics are summarized in Tables 1 and 2. This PACC3 BaBLR subset of WRAP participants differed from WRAP participants who did not meet inclusion criteria in the following ways: those excluded were slightly older at baseline, fewer had college degrees, and they scored lower, on average, on WRAT3 and AVLT (see Supplementary Table 1 for more details). Among the excluded individuals, 323 had PACC3 data, with 56.3% having one visit and 43.7% having two visits.

**Table 1:** Sample characteristics (demographics and cognitive measures), overall and by cognitive trajectory group

	Overall	Steep cognitive decline (*N* = 77)	Intermediate cognitive decline (*N* = 446)	Minimal cognitive decline (*N* = 545)	*P*-value^a^	Difference pairs
Demographics						
Age at cognitive baseline (years; median [IQR])	59.0 [53.7, 63.1]	63.3 [58.9, 66.2]	60.2 [54.6, 64.0]	57.3 [52.2, 61.2]	**<0**.**001**	**All pairs**
Age at most recent cognitive assessment (years; median [IQR])	66.9 [61.9, 71.6]	71.6 [68.0, 74.2]	68.0 [62.8, 72.4]	65.3 [60.8, 70.1]	**<0**.**001**	**All pairs**
Median follow-up duration (years)	8.34 [6.70, 9.99]	8.43 [6.69, 10.54]	8.02 [6.59, 9.50]	8.76 [6.76, 10.12]	**0**.**014**	**M versus I**
Gender/sex = female (%)	738 (69.1)	56 (72.7)	249 (55.8)	433 (79.4)	**<0**.**001**	**I versus S, M**
Family history = Y (%)	795 (74.4)	62 (80.5)	324 (72.6)	409 (75.0)	0.308	
College = Y (%)	677 (63.4)	47 (61.0)	242 (54.3)	388 (71.2)	**<0**.**001**	**M versus I**
APOE e4 carriers = positive (%)	407 (38.1)	46 (59.7)	161 (36.1)	200 (36.7)	**<0**.**001**	**S versus I, M**
WRAT3 reading (median [IQR])	109 [103, 115]	111 [105, 115]	106 [99, 113]	111 [105, 115]	**<0**.**001**	**I versus S, M**
Cognitive measures						
PACC3 baseline performance (median [IQR])	0.10 [−0.42, 0.58]	−0.05 [−0.41, 0.26]	−0.48 [−0.88, −0.19]	0.55 [0.28, 0.86]	**<0**.**001**	**All pairs**
PACC3 at most recent performance (median [IQR])	−0.02 [−0.62, 0.55]	−1.24 [−1.87, −0.99]	−0.50 [−0.92, −0.09]	0.51 [0.09, 0.90]	**<0**.**001**	**All pairs**
PACC3 baseline performance *Z*-score [lsmean (SE)]	0.056 (0.04)	−0.041 (0.05)	−0.54 (0.02)	0.48 (0.022)	**<0**.**001**	**All pairs**
PACC3 at most recent performance *Z*-score [lsmean (SE)]	−0.071 (0.05)	−1.43 (0.06)	−0.502 (0.03)	0.33 (0.03)	**<0**.**001**	**All pairs**
Self-reported memory (%)					**<0**.**001**	**All pairs**
No problem	717 (67.2)	38 (49.4)	282 (63.2)	397 (73.0)		
Neutral	237 (22.2)	20 (26.0)	105 (23.5)	112 (20.6)		
Major problem	113 (10.6)	19 (24.7)	59 (13.2)	35 (6.4)		

College = Y , education years ≥ 16. Bold values denote statistical significance at the *P* < 0.05 level.

^a^Statistical tests: *χ*^2^ or Fisher’s exact for categorical; ANOVA for continuous where M (SD) or lsmean (SE) reported; Kruskal–Wallis for continuous where median [Q1–Q3] reported and Likert-scale items. *Post hoc* pairwise group differences at BH-adjusted *P* < 0.05 noted in the right-hand column. For example, S versus I, M indicates group steep decline differed from group intermediate and group minimal decline in separate pairwise comparisons.

**Table 2:** Baseline health-related factors overall and by cognitive trajectory group

	Overall	Steep cognitive decline (*N* = 77)	Intermediate cognitive decline (*N* = 446)	Minimal cognitive decline (*N* = 545)	*P*-value^a^	Difference pairs
BMI (%)					0.078	
Normal	290 (27.2)	22 (28.6)	105 (23.6)	163 (30.0)		
Underweight	7 (0.7)	0 (0.0)	1 (0.2)	6 (1.1)		
Overweight	369 (34.6)	29 (37.7)	159 (35.8)	181 (33.3)		
Obese	399 (37.5)	26 (33.8)	179 (40.3)	194 (35.7)		
WHR (%)					**<0.001**	**I versus S, M**
Low	442 (41.6)	30 (39.0)	170 (38.3)	242 (44.6)		
Moderate	271 (25.5)	27 (35.1)	107 (24.1)	137 (25.3)		
High	350 (32.9)	20 (26.0)	167 (37.6)	163 (30.1)		
Prescription (%)					**<0.001**	**All pairs**
NP	791 (74.6)	44 (57.9)	318 (72.3)	429 (78.7)		
PP	228 (21.5)	25 (32.9)	105 (23.9)	98 (18.0)		
HP	42 (4.0)	7 (9.2)	17 (3.9)	18 (3.3)		
Hypertension (%)	346 (32.7)	33 (42.9)	152 (34.6)	161 (29.7)	**0.038**	**M versus S**
Diabetes (%)	90 (8.5)	12 (15.6)	42 (9.6)	36 (6.6)	**0.018**	**M versus S**
High cholesterol (%)	559 (52.8)	53 (68.8)	234 (53.3)	272 (50.2)	**0.009**	**S versus I, M**
Depression (%)	380 (36.0)	35 (45.5)	158 (36.1)	187 (34.6)	0.176	
Stroke (%)	20 (1.9)	2 (2.6)	5 (1.1)	13 (2.4)	0.317	
Heart (%)	256 (24.2)	20 (26.0)	108 (24.6)	128 (23.6)	0.873	
SRH (%)					**0.030**	**M versus I**
Excellent	607 (57.2)	41 (53.9)	234 (52.9)	332 (61.0)		
Good	394 (37.1)	27 (35.5)	182 (41.2)	185 (34.0)		
Poor	61 (5.7)	8 (10.5)	26 (5.9)	27 (5.0)		
LIBRA score tertiles (%)					**<0.001**	**M versus I**
Low	337 (31.6)	21 (27.3)	110 (24.7)	206 (37.8)		
Moderate	349 (32.7)	27 (35.1)	152 (34.1)	170 (31.2)		
High	382 (35.8)	29 (37.7)	184 (41.3)	169 (31.0)		

BMI, body mass index; HP, hyperpolypharmacy; LIBRA, LIfestyle for BRAin health; NP, no polypharmacy; PP, polypharmacy; SRH, self-rated health; WHR, waist-to-hip ratio. Bold values denote statistical significance at the *P* < 0.05 level.

^a^Statistical tests: *χ*^2^ or Fisher’s exact for categorical. *Post hoc* pairwise group differences at BH-adjusted *P* < 0.05 noted in the right-hand column. For example, S versus I, M indicates group steep decline differed from group intermediate and group minimal decline in separate pairwise comparisons.

### Cognitive trajectory groups

**Figure 1:**
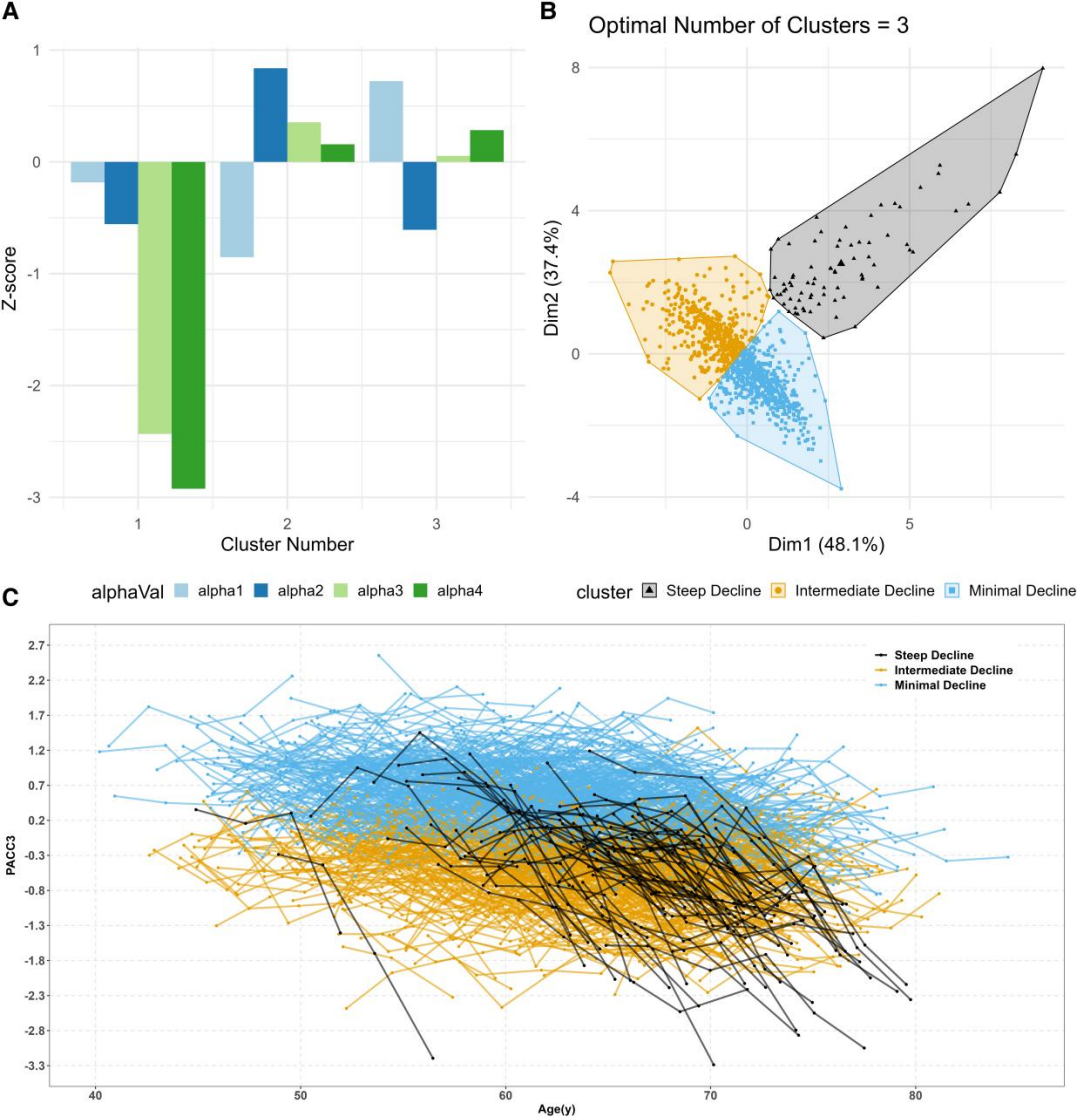
***K*-means clustering of participants’ cognitive trajectory.** (**A**) Mean *Z*-scores of each of the BaBLR random effects variables within each cluster number. The *Z*-scores are calculated for the current sample, yielding a sample sum of 0 and a standard deviation of 1; thus, the groups tend to approximately mirror each other around the *y* = 0 axis when the group sizes are similar. (**B**) Clusters are distributed along the principal components. Participants (*N* = 1068) are represented by points in the plot. (**C**) The spaghetti plot of PACC3 scores by cognitive trajectory groups PACC3—Preclinical Alzheimer’s Cognitive Composite (comprised of averaged *Z*-scores for three tests: AVLT Total Learning, Logical Memory Delayed Recall and Digit Symbol Substitution). Note: 88.2% CP has not been observed. 1.3% CP before the first observation and 10.5% have observed the CPs. Though not all subject’s CPs are observed, all have estimated slopes before and after their CP. These estimates, along with others from the model, are used as input for the cluster analysis. The optimal number of clusters given by the NbClust() function was 3. The Caliński and Harabasz value was 432.4. alpha1, posterior median estimate intercepts at CPs from 5000*4 iterations; alpha2, median estimate slopes pre-CP; alpha3, median estimate slopes post-CP; alpha4, median estimate person-level CPs for PACC3.

The scatter and correlation plots of the person-level BaBLR random effects are shown in Supplementary Figs 2 and 3. *K*-means cluster analysis using these random effects identified three cognitive trajectory clusters: Group 1, the smallest cluster, is characterized by a pattern of relatively average PACC3 performance at the CP with earlier CP and faster decline than the other clusters after the CP [‘steep decline’ group; *n* (%) = 77 (7.2%)]; Group 2 shows a pattern of generally low-average performance at the CP, later age of CP and weakening trajectories after CP [‘intermediate decline’ group; 446 (41.8%)]; and Group 3 showed high-average performance at CP and mild negative change after CP [‘minimal decline’ group; 545 (51.0%)]. As shown in Supplementary Table 2, the cognitive trajectory groups differ significantly in terms of individual random effects, except for the slope before CP between steep decline and minimal decline group. Average PACC3 baseline and most recent scores in each group are shown in Table 1, by group. There are substantial differences in baseline PACC3 scores (intermediate < steep < minimal cognitive decline) and last scores (steep < intermediate < minimal cognitive decline) among the three groups. In addition, the steep decline group had more self-reported memory problems. Figure 1 depicts differences in random effects across clusters (top left), the principal components (top right) and individual PACC3 performance versus age by cluster (i.e. spaghetti plot at the bottom).

Table 3 summarizes comparisons of PACC3 baseline and most recent consensus cognitive statuses across cognitive trajectory groups. The minimal group has 96.9% CU-S at PACC3 baseline and 96.5% CU-S at follow-up with the remainder CU-D for each. The intermediate group has 27% CU-D at PACC3 baseline and only 17% CU-D at follow-up, and this group has 2.7% MCI at both PACC3 baseline and last visit (remainder is CU-S for each), while the steep group showed a decrease in CU-S from 83.1% at baseline PACC3 to 31.2% at last PACC3 and increase from 2.6% MCI at PACC3 baseline and then 32.5% at last visit. Moreover, two people who had MCI at PACC3 baseline remained MCI at last visit in steep decline group. Within intermediate decline group, out of 12 people who had MCI at PACC3 baseline, eight people reverted to CU (six with CU-S and two with CU-D) while four people remained MCI. The median time from baseline CU status to first MCI or dementia status for those (*n* = 37) who have progressed to a clinical status in intermediate and steep decline group is 5.63 and 2.14 years, respectively.

**Table 3:** Differences in PACC3 baseline and most recent clinical characteristics overall and by cognitive trajectory group

	Overall (*N* = 1068)	Steep cognitive decline (*N* = 77)	Intermediate cognitive decline (*N* = 446)	Minimal cognitive decline (*N* = 545)	*P*-value^a^	Difference pairs
Baseline PACC3^b^ cognitive statuses by consensus conference (%)					**<0**.**001**	**All pairs**
CU-S	909 (85.1)	64 (83.1)	317 (71.1)	528 (96.9)		
CU-D	145 (13.6)	11 (14.3)	117 (26.2)	17 (3.1)		
MCI	14 (1.3)	2 (2.6)	12 (2.7)	0 (0.0)		
Most recent cognitive statuses by consensus conference (%)					**<0**.**001**	**All pairs**
CU-S	908 (85.0)	24 (31.2)	358 (80.3)	526 (96.5)		
CU-D	123 (11.5)	28 (36.4)	76 (17.0)	19 (3.5)		
MCI	33 (3.1)	21 (27.3)	12 (2.7)	0 (0.0)		
Dementia	4 (0.4)	4 (5.2)	0 (0.0)	0 (0.0)		
Years from baseline cognition to first visit with an MCI/dementia (median [IQR]) diagnostic^c^	5.12 [2.20, 7.67]	5.63 [4.77, 8.01]	2.14 [0.00, 5.16]	NA	**0**.**021**	**S versus I**

CU-S, cognitive unimpaired standard; CU-D, cognitive unimpaired decline; MCI, mild cognitive impairment. Bold values denote statistical significance at the *P* < 0.05 level.

^a^Statistical tests: *χ*^2^ or Fisher’s exact for categorical; Kruskal–Wallis for continuous where median [Q1–Q3] reported. *Post hoc* pairwise group differences at BH-adjusted *P* < 0.05 noted in the right-hand column. All pairs indicate group steep decline differed from group intermediate and group minimal decline, and group intermediate decline differed from group minimal decline in separate pairwise comparisons.

^b^All in this set were CU at study baseline; PACC3 baseline was Visit 2 for many.

^c^The sample size (*n* = 37) includes individuals who progressed to MCI or dementia after the baseline assessment.

### Demographic and baseline differences across cognitive trajectory groups

As shown in Tables 1 and 2, the cognitive trajectory groups did not significantly differ in terms of parental history of an Alzheimer’s disease dementia clinical diagnosis or BMI. Participants in the steep decline group were older, had more females and *APOE* e4 carriers, were less educated, had lower estimated premorbid ability (WRAT3 reading scores) and were generally less healthy than one or both other cognitive trajectory groups (for details, see Tables 1 and 2 and Supplementary Tables 3 and 4).

### Biomarkers by cognitive trajectory group

PiB, MK-6240, MRI scans and plasma occurred within mean (SD) 1.1 (2.7), 1.6 (1.4), 0.6 (0.3) and 0.09 (0.7) years of most recent cognitive assessment, respectively. Differences across groups are reported both by positivity status and continuous *Z*-scores.

#### Biomarker positivity

**Figure 2:**
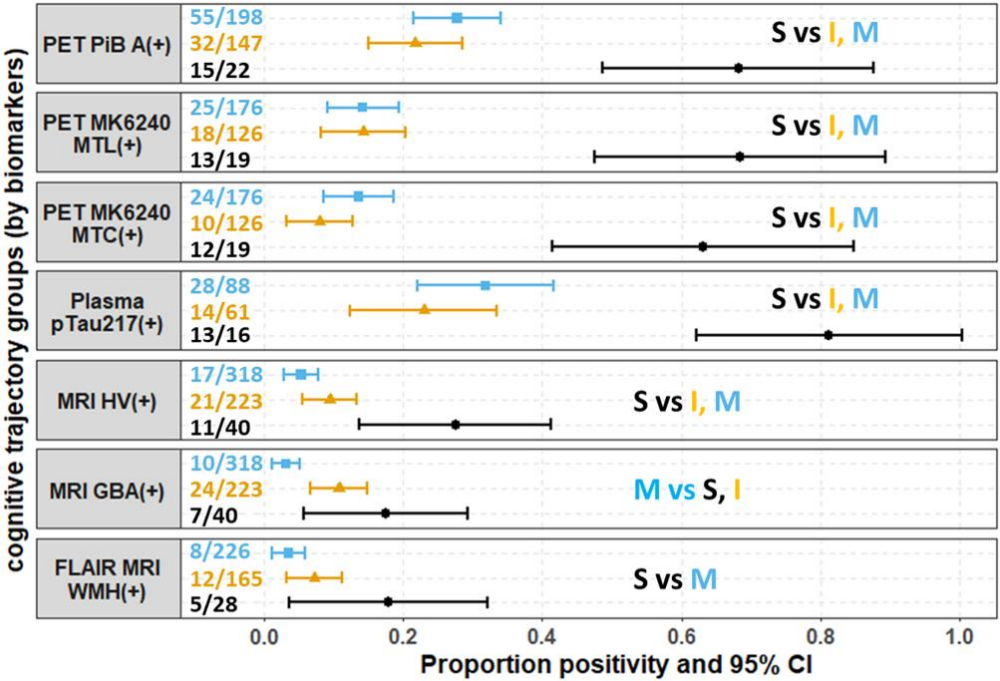
**Comparison of most recent dementia biomarker positivity across cognitive trajectory groups.** The positivity was defined in methods (primary). The number in the figure is the *n* of biomarker positivity in each cognitive trajectory group. Kruskal–Wallis tests were used. *Post hoc* pairwise group differences at BH-adjusted *P* < 0.05 noted in the text. For example, S vs I, M indicates group steep apparent cognitive decline group differed from intermediate and minimal decline group in separate pairwise comparisons. S, steep decline; I, intermediate decline; M, minimal decline; MTL, medial temporal lobe (early tau stage composite, based on an average of the entorhinal cortex, hippocampus and amygdala; Berron *et al*.^39^); MTC, meta-temporal composite (mid-to-late tau stage composite, based on Mayo MTC; Jack, Wiste, *et al*.^40^). HV, hippocampus volume; GBA, global brain atrophy; WMHV, white matter hyperintensity volume.

Figure 2 depicts biomarker positivity [using the primary (*Z*-score) method to define positive] by cognitive trajectory group and biomarker (*P* < 0.05 for all cognitive trajectory group omnibus tests). In *post hoc* comparisons, the steep decline group had more biomarker positivity than one or both other groups for all biomarkers. Although the secondary method of defining biomarker positivity identified higher proportions of pTau217+, HV+, GBA+ and WMHV+, *post hoc* pairwise group differences were ostensibly the same, except the steep decline group also differed significantly from the intermediate group in WMHV positivity (Supplementary Fig. 4).

#### Continuous biomarkers

**Figure 3:**
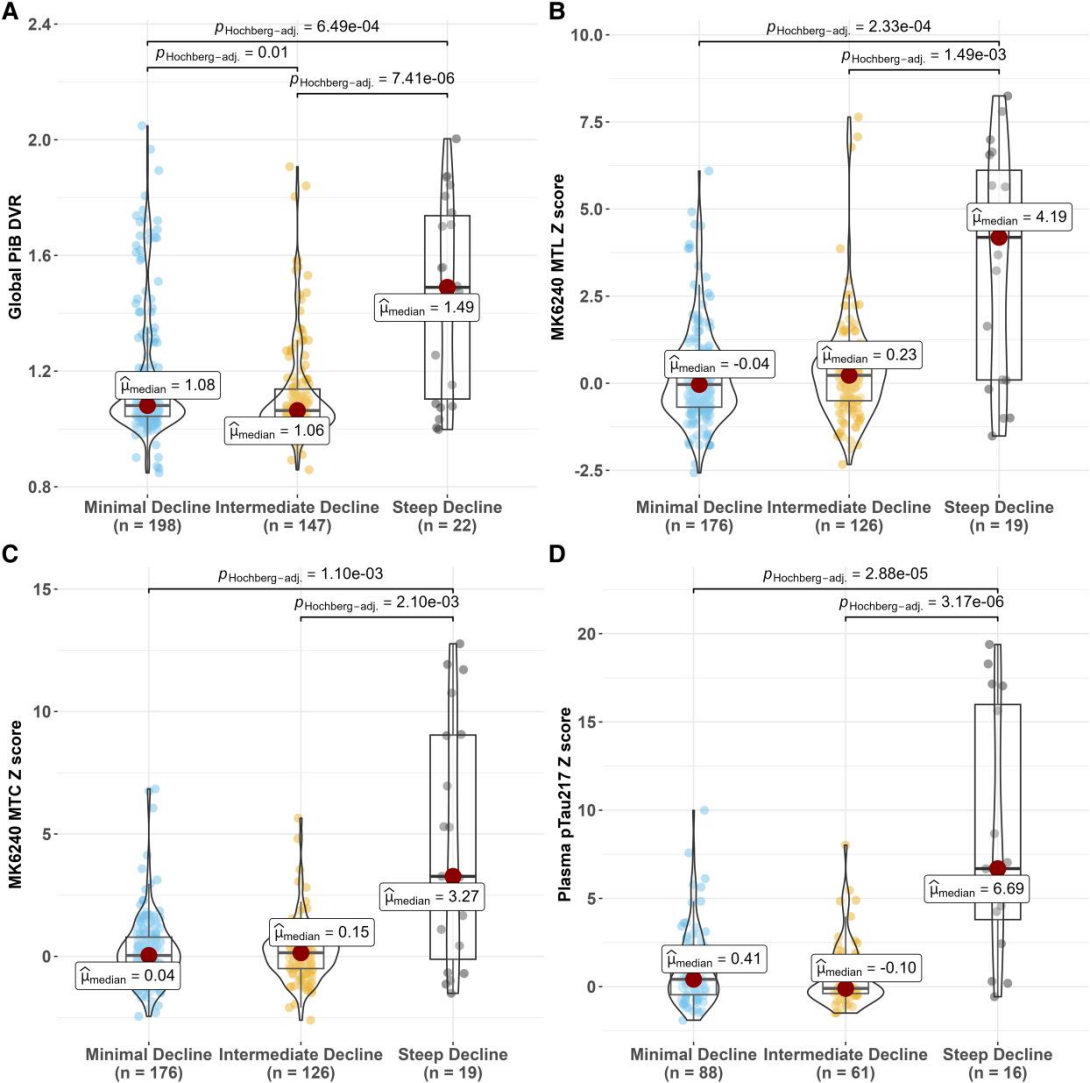
**Comparison of most recent PET and plasma biomarkers across cognitive trajectory groups.** (**A**) PET global PiB DVR, (**B**) MK-6240 MTL composite *Z*-score, (**C**) MTC *Z*-score and (**D**) plasma pTau217 *Z*-score. We used the PiB+ cut-off of 1.16 to identify a CU, PET A- subset for each biomarker/variable with the plan to create *Z*-scores using the mean (SD) of that group (from the last value available biomarker measure for each person in that CU, A- subset). Statistical tests: Kruskal–Wallis test was conducted to examine the biomarker differences on PACC3. Significant differences (global PiB DVR: *χ*^2^ = 23.7, *P* < 0.0001, df = 2; MK-6240 MTL: *χ*^2^ = 15.7, *P* < 0.001, df = 2; MK-6240 MTC: *χ*^2^ = 12.8, *P* < 0.01, df = 2; plasma pTau217: *χ*^2^ = 24.2, *P* < 0.0001, df = 2) were found among the cognitive trajectory groups of participants. *Post hoc* significant pairwise group differences at BH-adjusted method *P* < 0.05 are shown in the figure. The larger dots stand for the mean value in each profile. The effect sizes are summarized in Supplementary Fig. 5 and Table 5. CU, cognitive unimpaired; A-, amyloid PET negative.

**Figure 4:**
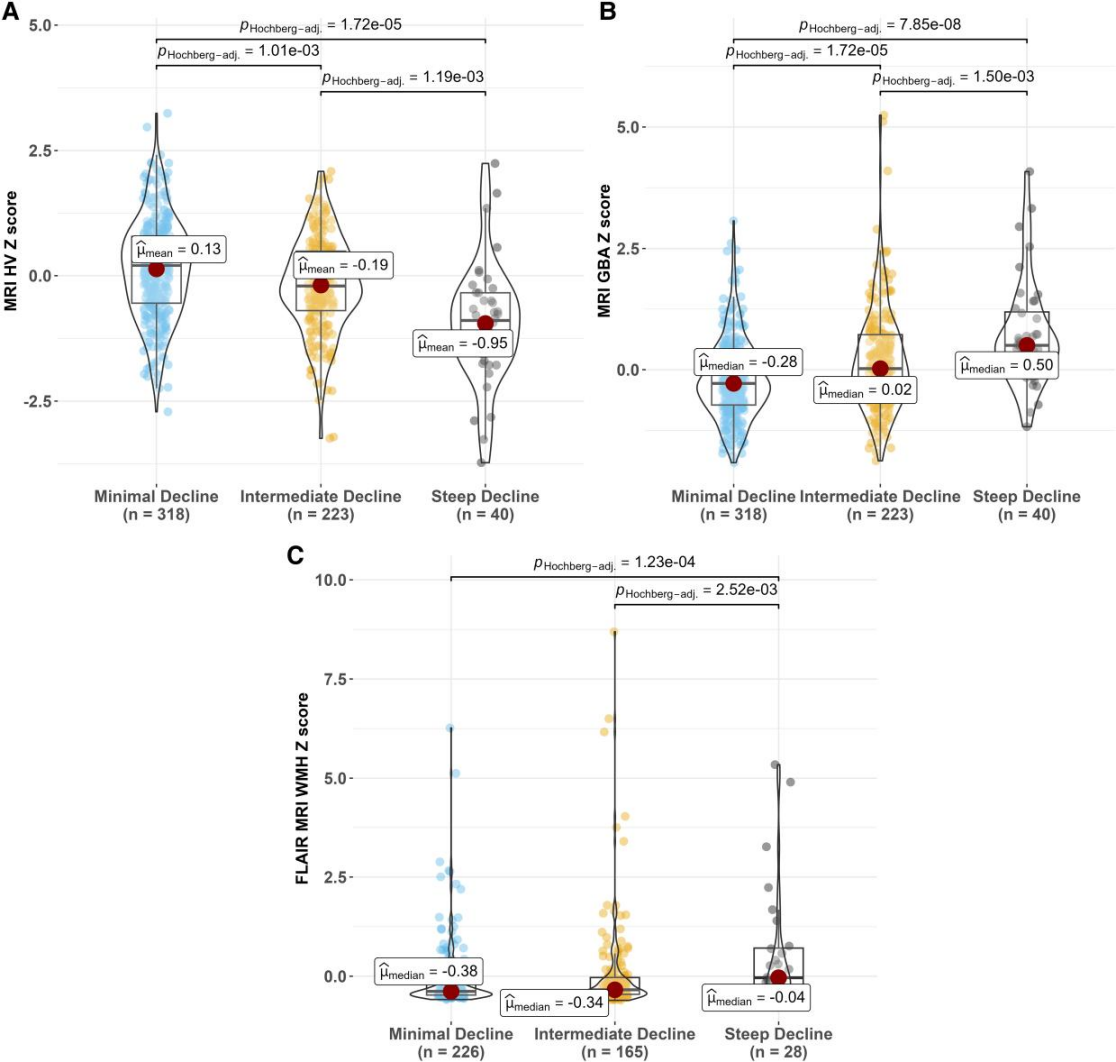
**Comparison of most recent MRI biomarkers across cognitive trajectory groups.** (**A**) MRI HV *Z*-score, (**B**) MRI GBA *Z*-score and (**C**) WMHV *Z*-score. To get the MRI biomarker regression-based *Z*-scores (i) using the CU, PET A- subset, we ran the regression equation: biomarkers = intercept + beta*TICV, and saved the beta’s and RMSE from this equation. (ii) For everyone with the biomarkers, we used the regression equation to get predicted biomarkers and calculated the *Z*-score as follows: (Observed biomarkers − Predicted biomarkers)/(Root mean squared error from CU, A- subset regression model that adjusted for TICV). Statistical tests: ANOVA for MRI HV *Z*-score; Kruskal–Wallis for other continuous variables. Significant differences (MRI HV: *F*(2,104.5) = 18.7, *P* < 0.0001; MRI GBA: *χ*^2^ = 42.1, *P* < 0.0001, df = 2; FLAIR MRI WMH: *χ*^2^ = 17.4, *P* < 0.001, df = 2) were found among the cognitive trajectory groups of participants. *Post hoc* significant pairwise group differences at BH-adjusted method *P* < 0.05 are shown in the figure. The red dots stand for the mean value in each profile. The effect sizes are summarized in Supplementary Fig. 5 and Table 5. CU, cognitive unimpaired; A-, amyloid PET negative; TICV, total intracranial volume; RMSE, root mean square error.

All omnibus tests of continuous biomarkers showed significant differences across cognitive trajectory groups (*P* < 0.001; Figs 3 and 4). *Post hoc* comparisons showed that the steep decline group had worse MK-tau, pTau217, HV, GBA and WMHV than both the intermediate and minimal decline groups and worse PiB than the minimal decline group. In addition, the intermediate decline group had lower PiB but worse HV, GBA and WMHV than the minimal cognitive decline group. In the A+ subset (*n* = 101), EAOA was significantly younger in the steep decline group than both other groups (omnibus *P* = 0.024). Median EAOA (Q1, Q3) for the cognitive trajectory groups were as follows: steep decline, 54.7 (47.4, 60.4); intermediate, 64.1 (57.9, 70.1); and minimal, 61.0 (54.1, 66.7). In the sensitivity analysis adjusting for covariates (age, sex), the previously detected differences among steep and intermediate, steep and minimal decline groups remained significant in PiB-amyloid, MK-tau, pTau217 and HV models; the difference between intermediate and minimal groups showed significance in MK-tau and pTau217 and remained significant in WMHV, but resultant models of GBA did not differ by cognitive trajectory group. The effect sizes with and without adjusting covariates are summarized in Supplementary Table 5 and Fig. 5.

## Discussion

In this large group of initially cognitively unimpaired late middle-aged adults, we first characterized the individual prospective PACC3 cognitive trajectories over a span of up to 13 years with a newly developed BaBLR method for characterizing longitudinal within-person patterns.^19^ Three cognitive trajectory groups were then identified using cluster analysis of the random effects extracted from this model (CP, slope before CP, slope after CP and estimated PACC3 at CP). The smallest group had the worst trajectories (steep cognitive decline, ∼7%) while the largest group had the best trajectories (minimal decline, 51%). The relationships of the identified latent cognitive trajectory groups were then examined in relation to a comprehensive and diverse demographic, health-related factors and Alzheimer’s disease and other dementia-related biomarker measurements. Our findings indicated that worse status and/or abnormal values on measures of general health and brain integrity were consistently associated with the group showing the most rapid preclinical decline relative to the other cognitive trajectory clusters. Therefore, the three identified clusters represent distinct groups, and certain people are categorized as either a steep/intermediate/minimal decliner. The higher proportion of abnormalities on other dementia-related biomarkers suggest that individual heterogeneity in cognition can also be connected to preclinical decline towards non-Alzheimer’s disease dementia and/or multi-aetiology dementia.

### Cognitive trajectory groups

The three cognitive trajectory groups differed in several ways. The median last PACC3 scores were much lower than baseline in the steep decline group [changing from a median *Z*-score of −0.04 at baseline to −1.43 at last PACC3 (Table 1) corresponding to decline from the 41.8 percentile to 5.2 percentile]. In contrast, the minimal decline group dropped from a baseline median *Z*-score of 0.48–0.33 (Table 1) corresponding to only a decline of 2.3 percentiles. The intermediate decline group declined slower than the other two groups (changing from a median *Z*-score of −0.48 at baseline to −0.49 at last PACC3). Both the intermediate and steep decline groups were older and contained proportionally greater numbers of participants who had more CU-D, MCI or dementia at most recent visit, with 68.9% of the steep decline group having a status of CU-D, MCI or dementia, relative to only 3.5% CU-D of the minimal decline group (Table 3). The median time from baseline CU status to first MCI or dementia status for those (*n* = 37) who have progressed to a clinical status was shorter in intermediate decline group (5.63) than steep decline group (2.14). This difference might be attributed to the fact that Group 2 exhibited worse performance (lower PACC3) at baseline. The steep decline group had faster decline than the other clusters after the CP. This supports that the preclinical change is happening well before the onset of clinical dementia.^53,54^ The minimal decline group showed high average performance at CP and mild negative change after CP. This is consistent with the results of prior studies,^55–57^ which have shown that in the absence of preclinical neurodegenerative disease, episodic memory, semantic memory and processing speed show only very subtle or no decline in cognitively normal (CN) older adults. These results suggest that cognitive decline is minimal in healthiest agers (minimal decline group).

The identification of three different cognitive trajectories is consistent with results of a multi-centre prospective cohort study of 333 CN older adults [mean (SD) age 70 (6.8)] assessed over a 54-month period.^58^ In that study, most (65.5%) of the sample showed an above average and stable memory trajectory, whereas 30.9% showed a below average and subtly declining memory trajectory and 3.6% showed a below average and rapidly declining memory trajectory using latent growth mixture modelling.^58^ The sample in Pietrzak *et al*.’s^58^ study was older on average, smaller and had shorter follow-up. In addition, their analysis method did not allow slopes and intercepts to vary across participants. In a larger study of 19 114 CN older adults [age 65+ years (Black and Hispanics) or 70+ years (all other ethno-racial groups)] whose cognition was followed prospectively for up to 7 years, four to seven trajectories were identified per cognitive domain using GBTM.^59^ In that study using methods similar to Pietrzak *et al*.’s to identify cognitive subgroups, substantial decline in global cognition and episodic memory were observed in a small proportion of individuals.^59^

### Demographic and baseline health-related differences across cognitive groups

Overall, the multiplex of factors that differed between the cognitive decline groups extends results reported previously using WRAP data. For example, one study reported that the presence of two lifestyle/modifiable risk factors (hypertension or obesity) in midlife exacerbated subtle cognitive decline in a sample of 207 late middle-aged adults who had completed 3+ neuropsychological evaluations and a [^11^C]PiB PET scan or lumbar puncture.^60^ Another study in initially cognitively unimpaired, late middle-aged participants (*N* = 1215; baseline mean age 59.3 years) found lower baseline LIBRA index, denoting healthier lifestyle and lower dementia risk, was related to better overall cognitive performance.^5^ In the current study using updated WRAP data, one or both of the more declining groups (steep and intermediate) differed from the minimal decline group in the following ways: higher chronological age, lower education, lower estimated premorbid functioning, greater prescription drug use, increasing number of medical comorbidities, increased self-reported depression, worse LIBRA scores, greater proportion of *APOE* e4 carriage, poorer SRH and greater subjective cognitive difficulties. These findings are consistent with risk factors for dementia identified in a review by The Lancet International Commission on Dementia Prevention and Care by synthesis of wide ranging group data.^61^ Wu *et al*.^21,62^ also found that participants in lower-functioning classes were older, with notably fewer years of education and had an increased risk of subsequent physical disability, dementia or mortality compared to those who maintained high cognitive performance. Another study examined the trajectories of cognitive decline across the late-life period in a large cohort of elderly participants and showed participants within the ‘no decline’ class were significantly more likely, relative to the other three cognitive groups, to report healthy lifestyle (e.g. exercise).^11^ The samples from these other cohorts were older and had shorter follow-up than the WRAP cohort. In addition, our analysis methods leveraged within-person cognitive patterns rather than relying on group averages as previous clustering approaches have done.

Several studies have noted the following paradoxical pattern. Namely, among cognitively healthy individuals, women generally outperform men on cognitive assessments at older ages; however, women have also been found to be at higher risk of Alzheimer’s disease than men.^63–65^ Our results showing more women in both the steep and minimal decline groups (relative to the intermediate group) are consistent with these findings. The patterns of higher BMI and WHR observed in our intermediate cognitive decline group are consistent with other studies that show that being overweight or obese in midlife may be more detrimental to subsequent age-related cognitive decline^66^ while a different study (mean age 75) suggested a potentially protective effect of obesity on Alzheimer’s disease in the elderly.^67^

### Biomarker differences across cognitive groups

We hypothesized that a pattern depicting early CP, lower score at CP and faster decline in PACC3 would be associated with worse status on A/T/N/V biomarkers representing brain health.

The Alzheimer’s disease brain is characterized pathologically by the combined presence of two classes of abnormal structures, extracellular amyloid plaques and intraneuronal neurofibrillary tangles, both of which comprise insoluble, densely packed filaments.^68^ The results here are in agreement with our previous findings in a smaller sample of initially cognitively unimpaired participants; in that analysis, those with both elevated biomarkers for pathological amyloid-β and tau declined faster than those with just one or no elevated biomarkers.^6^ In our study, the steep decline group had more pTau217+ than both other groups, which is consistent with a recent study of WRAP plasma sample that showed higher baseline pTau217 was associated with worse cognitive trajectories. In addition, among those who were PET A+, the steep decline group had earlier amyloid onset age. Our previous studies in smaller samples have found that higher chronicity (amyloid duration) was associated with faster cognitive decline and increased risk of pre-clinical or clinical decline.^7^ Similar relationships between amyloid-β, tau, pTau217 and cognition have been found in several other studies of cognitively unimpaired subjects.^39,40,44,58,69–73^ For example, prospective studies indicate that CN older adults with biomarker evidence of Alzheimer’s disease neuropathology show subtle progressive memory decline.^73^ Our report is unique from these previous studies in that we established such relationships with biomarkers measured in late midlife (i.e. at earlier ages than most other studies).

The current work adds to the growing number of recent studies examining the association between neurodegeneration biomarkers and cognition in cognitively unimpaired subjects.^74–76^ A prior study^74^ with CU late middle-aged adults from WRAP and Wisconsin ADRC found that individuals with GBA had greater verbal learning and memory decline than those without but no significant association between HV and cognition. However, the smaller sample size (*N* = 342) and use of raw biomarker variables instead of *Z*-scores and categorical biomarker groupings may account for this discrepancy. Our finding is consistent with the established literature,^77–80^ which suggests that WMH are often associated with cognitive decline. Nettiksimmons *et al*.^79^ found that a subgroup with substantial brain atrophy and WMH had a worse cognitive trajectory. In our previous study,^45^ individuals were grouped based on biomarkers of amyloid pathology, MRI-derived measures of neurodegeneration/atrophy, neurofibrillary tangles and a brain-based marker of vascular risk. Four biomarker clusters emerged, and three of them exhibited decline on different cognitive functions.

Taken together, these results suggest that A/T/N/V biomarkers associate with cognition decline. Our previous study found WMH were associated with decreasing executive functioning and speed of processing but not memory.^77^ These results suggest that selecting sensitive neuropsychological tests to detect the preclinical changes is important. Composite cognitive tests are being used increasingly as primary endpoints in Alzheimer’s disease trials, particularly preclinical trials since traditional outcome measures are not sensitive to tracking preclinical cognitive decline.^81^ Mormino *et al*.^82^ showed individual PACC components varied in their ability to measure cognitive decline throughout the continuum of preclinical Alzheimer’s disease. The work by Langbaum *et al*.^81^ has shown that a combination of six to seven tests spanning perceptual speed/working memory, orientation, episodic memory and visual spatial is sensitive to preclinical Alzheimer’s disease decline up to 11 years prior to diagnosis of the clinical stages of Alzheimer’s disease. The WRAP cognitive composite PACC3 has been shown to be sensitive to preclinical Aβ burden.^7,82,83^ This composite score has two or three tests in common with the late-onset Alzheimer’s Prevention Initiative Preclinical Cognitive Composite^81^ as well as the ADCS PACC^22^ but omits mini-mental state examination, due to its limited sensitivity in middle-aged predominantly healthy samples.^82^ Our finding suggested that within-person PACC3 performance patterns are sensitive to preclinical changes that are associated with A/T/N/V biomarkers. Trials that aim to prevent cognitive impairment should use outcome measures that are sensitive enough to capture early cognitive changes and are preferably also good surrogates for future development of dementia. No gold standard of outcome measures currently exists, but composite scores that include several cognitive tests are recommended. Our study showed PACC3 could be a good outcome measure for early cognitive changes. The low overall PACC3 performance combined with the higher proportion of abnormalities on health variables of the intermediate group suggests that lifestyle interventions earlier in life may preserve cognitive performance. The Protect Brain Health through Lifestyle Intervention to Reduce Risk (POINTER) Study^84^ and the Finnish Geriatric Intervention Study to Prevent Cognitive Impairment and Disability (FINGER) Study^85^ have also shown that it is possible to prevent cognitive decline using a multi-domain lifestyle intervention. Also, early initiation of interventions, thus targeting younger and healthier individuals than those targeted by previous trials, might lead to better results. And focusing prevention trials on at-risk individuals might be an effective and feasible approach.

### Strengths and limitations

Our analyses have several strengths. Firstly, we initially performed a framework combining the BaBLR model and cluster analysis to identify longitudinal trajectories of cognitive performance. The BaBLR model allowed for individual random effects^19^ and may inform preventative strategies with person-level estimates. The *K*-means clustering approach allowed for more data-driven trajectories without preset parameters. Secondly, our moderately large longitudinal cohort from midlife, initially cognitively unimpaired, allowed for robust estimation of heterogeneity in preclinical changes. We also examined a comprehensive set of health-related measures and biomarkers across cognitive trajectory groups. Another strength is that we used a unified approach to get cut-offs for T/N/V biomarkers using the CU/A- subset to generate *Z*-scores. However, some biomarkers may not be accurate for Alzheimer’s disease, and only one cognitive composite was considered. In future analyses, we will apply the CP modelling to a larger set of cognitive outcomes and address additional research questions related to them. For example, while the PACC3 has been shown to be sensitive to preclinical change, changes may be apparent earlier in some of the tests contributing to it or in other tests. Previous work has attempted to disentangle when changes are occurring across cognitive tests (for example, see Bilgel *et al*.^86^). The BaBLR method may allow us to get more precise understanding of which cognitive composites or tests change earliest during preclinical changes towards dementia and which are likely to hit levels associated with clinical impairment fastest. In addition, differences in Supplementary Table 1 suggest that some steep and intermediate decliners might not be included in the study because only people with at least three PACC3 visits were included. Future refinements to the BaBLR method will seek to accommodate those with only one or two data points to reduce potential selection bias. Furthermore, the WRAP cohort is largely white and well educated. Future studies should validate these results across multiple cognitive domains in more diverse cohorts.

## Conclusion

In this initially cognitively unimpaired sample from late midlife, differences between identified cognitive trajectory groups across multiple risk factors and dementia biomarkers indicated that within-person PACC3 performance patterns were sensitive to preclinical change and, most importantly, those who are showing early decline carry multiple indicators of worse physical and brain health. Identifying cognitive trajectory clusters may provide an opportunity for early intervention for high-risk subjects at the right time for treatment and/or enrolment in a clinical trial.

## Supplementary Material

fcad333_Supplementary_Data

## Data Availability

Data from the WRAP cohorts can be requested through an online submission process (https://wrap.wisc.edu/data-requests/).
