# Supplementary material for: Harnessing cognitive trajectory clusterings to examine subclinical decline risk factors
Source: Brain Commun. 2023 Dec 3;5(6):fcad333. doi: 10.1093/braincomms/fcad333 (PMC10724051; doi:10.1093/braincomms/fcad333)
Supplement: fcad333_Supplementary_Data [file fcad333_supplementary_data.docx]

**Supplementary material**

**Supplementary Materials Overview**

**Supplementary materials are divided into three broad parts. Part 1 includes details augmenting what is reported in the “Biomarker Positivity Methods” paragraph in the Methods section. Part 2 includes more in-depth explanation of the BaBLR method that is described in the “Statistical Analyses” portion of the Methods section. Finally, Part 3 includes supplementary tables and figures that are referenced in the Results section.**

**Part 1: Supplementary Biomarker Positivity Methods**

The purpose of this section is to document our approach to identifying biomarker thresholds used in the manuscript entitled, *“Harnessing cognitive trajectory clusterings to examine subclinical decline risk factors”*. Specifically, we completed these analyses after consultations with a clinical neuropsychologist and scientists in our group on various options for determining cut-offs; while several options exist, we focused on a primary and secondary method. In the primary approach, we transformed each of the biomarkers to a z-scale relative to the PET A-, CU subsample and used a set threshold of 1.5 (or -1.5 when lower is worse) to identify abnormal for each biomarker. In the secondary approach, we used Gaussian Mixture Model (GMM) with each biomarker to identify a threshold. To cross-check the thresholds, we obtained using our primary and secondary methods, we ran Receiver-Operator Characteristic (ROC) analyses of the biomarker z-scores relative to PET A-/+ status to identify thresholds. Details follow.

**Step 1.** Create z-scores for each biomarker/measure including: MK6240 MTL composite and MTC composite; plasma pTau 217, MRI Hippocampal volume (HV), global brain atrophy (GBA), and white matter hyperintensities (WMH).

**Step 1a)** We identified a reference group of CU, PET A- participants in the sample using a cut-off of Global PiB DVR=1.16 (corresponding centiloid of 17.7, previously shown to predict accumulation, (Betthauser et al., 2022; Farrell et al., 2021)) to identify the CU, PET A- subset for each biomarker/variable. This threshold is slightly lower than a published threshold used by WRAP (1.19); (Racine et al., 2016)); the lower threshold is less likely to include subthreshold accumulators in the CU, A- reference group.

**Step 1b)** For biomarkers that did not require covariate adjustment, we then determined the mean (sd) of the last value available in the CU, PET A- subset and used these values to create z-scores as shown in Supplemental Table S4. These biomarkers included the MK6240 ROI’s Medial temporal lobe (MTL) composite and Meta-temporal composite (MTC) and plasma pTau217 data. For variables that did include covariate adjustment (e.g., adjusting HV for TICV), we used a regression-based approach based on Malone et al.’s (Malone et al., 2015) analyses showing that ANCOVA and residual approaches are the preferable approach for adjusting for TICV compared with dividing regional brain volumes by TICV (known as the proportion method). Supplementary Table 6 summarizes the n’s of the CU, PET A- subset for each biomarker, the equation used, and the parameter values used. While we used the regression-based approach in our analyses for the primary method of calculating WMH z-scores, we also calculated the z-score for WMH a second way that is also commonly used in literature. Namely, we also created the z score for the WMH/TIV ratio, where z-score is the

(Observed WMH/TIV value – Mean value of CU, PET A-)/(Standard deviation of CU, PET A- ). We include a scatter plot for regression-based and ratio-based z-scores in Supplementary Figure 6.

The red solid line is the diagonal line. Quadrant plots indicating the observed relationship between WMH regression-based and ratio-based z-scores are shown in Figure with positivity thresholds (z = 1.5 approach) for these two z-scores indicated with blue dashed lines. To get WMH regression-based z scores: 1) using the CU, PET A- subset, we ran the regression equation: WMH = intercept + beta*TICV and saved the beta’s and RMSE from this equation. Then for everyone with an WMH, we used the regression equation to get predicted WMH and calculate the z-score as: (Observed WMH – Predicted WMH)/(Root mean squared error from CU, A- subset regression model that adjusted for TICV). The equation to get WMH ratio-based z score was (Observed WMH/TIV value – Mean value of CU, PET A-)/(Standard deviation of CU, PET A-)

**Step 2**. Use the z-scores to define the thresholds

We used two methods for each biomarker to identify thresholds: In the primary method, we used the same threshold of z=1.5 (or -1.5 if low is abnormal) as the cut-off across all biomarkers. In the secondary method, thresholds were identified using GMM of the z-scores for each biomarker. When using z=1.5 as the threshold, our classification was consistent with levels determined by co-authors for overlapping samples (e.g., Cody et al, (*In review*) for MK6240 MTL and Jonaitis et al (Jonaitis et al., 2023) for ptau217 relative to MK-tau).

Supplementary Figure 7 depicts the thresholds by the z = 1.5/-1.5 approach (blue dotted line) and by GMM (red dotted line).

The dots are colored by the classification using the threshold described by Cody et al, (*In review*) (the 95%ile of the CU Aβ-negative (A-) participants) for MK6240 and the threshold that was defined by Jonaitis et al (ROC curve relative to MK tau+) for plasma pTau217. For HV, GBA, and WMH, the dots are colored by Gender/Sex.

Supplementary Table 7 summarizes the biomarker positivity proportions using the various approaches.

Previously, we indicated that we used z-score cut-offs of 1.5 or -1.5 as our primary method of identifying abnormality across the set of biomarkers we examined in this study and GMM as our secondary method. Receiver-Operator Characteristic (ROC) curves are also often used to establish cut-offs. To cross-check the thresholds we obtained using our primary and secondary methods, we ran ROC analyses of the biomarker z-scores relative to PET A-/+ status (where PET A+ was defined as a Global PiB DVR >1.16 and >1.19; both thresholds have been identified in the WRAP sample as reliable thresholds for amyloid positivity) to identify thresholds. Thresholds were selected with respect to maximize Youden’s index. Using this approach, the positivity rates were yielding positivity proportions that were clinically unlikely (and much higher than those based on z-score cut-offs of 1.5 or -1.5 or GMM thresholds) and not very high AUC’s for some of the measures. Supplementary Figure 8 - Supplementary Figure 13 showed the ROC’s for completeness/sphere of reference. The order of ROC analyses presented is: MK6240 MTL, MK6240 MTC, Plasma ptau217, HV, GBA and WMH. Not surprisingly, ROC analyses using PET A status had higher AUC’s for the AD-related A/T biomarkers than for the MRI based measures of neurodegeneration and cerebrovascular disease.

**Part 2: Supplementary Bayesian Bent-Line Regression (BaBLR) methods**

Bayesian Bent-Line Regression with a random CP model for longitudinal data involves four random effects (CPs, pre-change slopes, the differences between pre- and post-change slopes, and the expected response at CPs), which are modelled to vary among individuals around central population values. We do not constrain the slope before the CP since its pattern varies. However, the difference between pre- and post-change slopes is constrained to be negative, as we expect a more precipitous decline following the CP. This approach may be used to analyze subtle longitudinal change; it is a flexible technique that is appropriate to use to study heterogeneity in change patterns.

Below is the form of the BaBLR model.

$y_{ij}=\beta_{1i}+\beta_{2i}\left( t_{ij}-\omega_{i} \right)I\left( t_{ij}\leq\omega_{i} \right)+\left( \beta_{2i}+\beta_{3i} \right)\left( t_{ij}-\omega_{i} \right)I\left( t_{ij}>\omega_{i} \right)+\varepsilon_{ij}$ (1)

Where $y_{ij}=y_{ij}\left( t_{ij} \right)$ denote the *j*th observed continuous outcome measurement taken for the $i$th participant $(i=1,\cdots, N)$ at some time points $t_{ij}$ $(j=1,\cdots, n_{i})$ where $n_{i}$ is the number of measurements for subject $i$ and $N$ is the number of subjects, $I$ is the indicator function, $\omega_{i}$ is the individual-specific CP, $\beta_{1i}$ is the individual-specific intercept denoting the expected value of the cognitive test score at the CP, $\beta_{2i}$ is the individual-specific linear slope before the CP (pre-change slope), and $\beta_{3i}$is the individual-specific difference between pre- and post-change slope difference). We choose to model the difference in slopes instead of the post-slope directly to impose a sign constraint on this difference. We assume that the slope decrement $\beta_{3i}$ is equal to or less than or zero, so that cognition declines more rapidly after the CP than before. It's worth noting that the model permits cognition to potentially increase after the CP, albeit with a smaller slope than before, although we anticipate that most individuals will show an actual negative slope. The term $\varepsilon_{ij}$ accounts for individual measurement noise.

Calculating the maximum likelihood estimator while adhering to constraints is frequently challenging. Therefore, we opt for Bayesian methods in parameter estimation, as order constraints can be easily integrated into the prior distributions of the model parameters. The BaBLR model is fitted using Hamiltonian Monte Carlo (HMC) through the freely available software Stan. This approach dynamically refines the sampling process during a warm-up phase and proficiently samples from posterior distributions containing correlated parameters - often encountered in the cognitive model. It supports both built-in and user-defined distributions.

The simulation studies aimed at assessing the finite sample performance of the proposed model demonstrate that both estimation and inferential procedures exhibit reasonable performance with limited sample sizes. To illustrate practical use, we applied the model to a longitudinal cognitive composite PACC3 in the Wisconsin Registry for Alzheimer's Prevention (WRAP).

However, it's important to exercise caution with respect to sensitivity to prior distribution selection, identifiability concerns, and goodness of fit. In this context, two sensitivity analyses were conducted to gauge the potential impact of priors. First, we examined different half-normal, log-normal, and Student's t distributions to elucidate our choice of prior and its advantages. Second, we explored alternative priors for the mean of the changepoint (CP) to evaluate the influence of priors on CPs.

Furthermore, validation was undertaken for the BaBLR model. Half of the individuals were randomly chosen, leaving out their last observations, in order to determine how many of these last observations fell within the 0.95 quantile of the predicted interval. The analyses suggested the BaBLR model did well at predicting next visit performance.

Please see the details in Du et al. 2022 (Du et al., 2022).

**Part 3: Supplementary tables and figures**

**
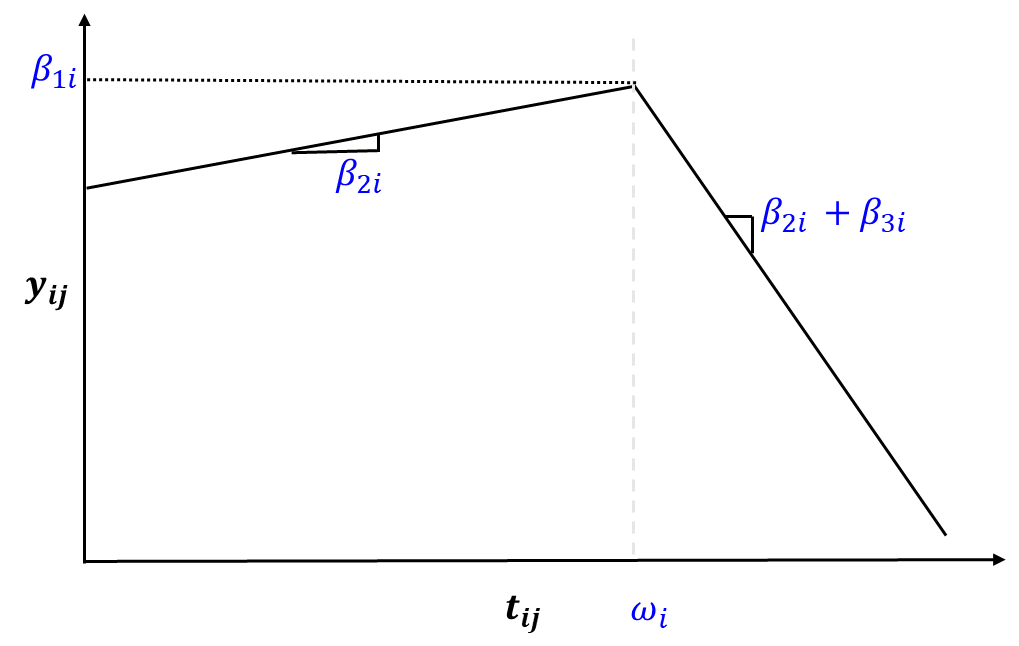
**

**Supplementary Figure 1 Bayesian Bent-Line Regression (BaBLR) model illustration plot.**

The parameters in this figure have the following interpretation: $t_{i}$ is time scale (age in years), y is the outcome (PACC3), $\omega_{i}$ is the individual-specific CP, $\beta_{1i}$ is the individual-specific intercept denoting the expected value of the cognitive test score at the CP, $\beta_{2i}$is the individual-specific linear slope before the CP (pre-change slope), and $\beta_{3i}$ is the individual-specific difference between pre- and post-change slope difference).


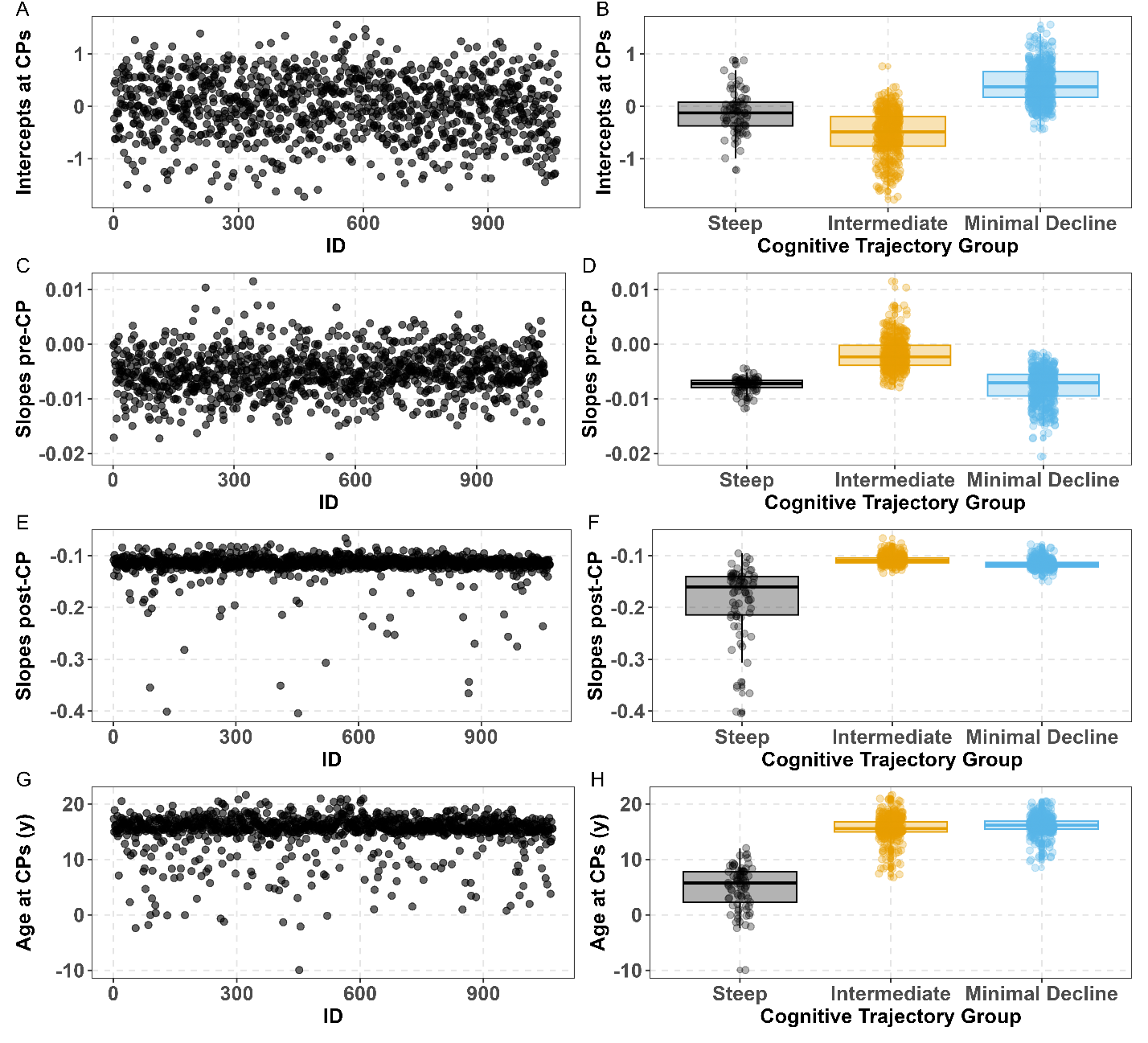


**Supplementary Figure 2** **The scatter and boxplot of individual random effects**

The random effects (alpha1: posterior median estimate intercepts at CPs from 5000*4 iterations; alpha2: median estimate slopes pre-CP; alpha3: median estimate slopes post-CP; and alpha4: median estimate person-level CPs for PACC3) were extracted from Bayesian Bent-Line Regression model using the longitudinal PACC3 data. PACC3 = Preclinical Alzheimer’s Cognitive Composite (comprised of averaged z-scores for 3 tests: Auditory Verbal Learning Test (AVLT) Total Learning, Logical Memory Delayed Recall, and Digit Symbol Substitution). The left-hand column (A, C, E, G) is the scatterplot of each individual random effects. The x axis represents the individual number, and the y axis represents the random effects (coefficients). The right-hand column (B, D, F, H) shows boxplots of each individual random effects, by cognitive trajectory groups. To map on to the parameters we defined in the Supplementary Figure 1: alpha1 = $\beta_{1i}$, alpha2 = $\beta_{2i}$, alpha3 = $\beta_{3i}$, alpha4 = $\omega_{i}$.


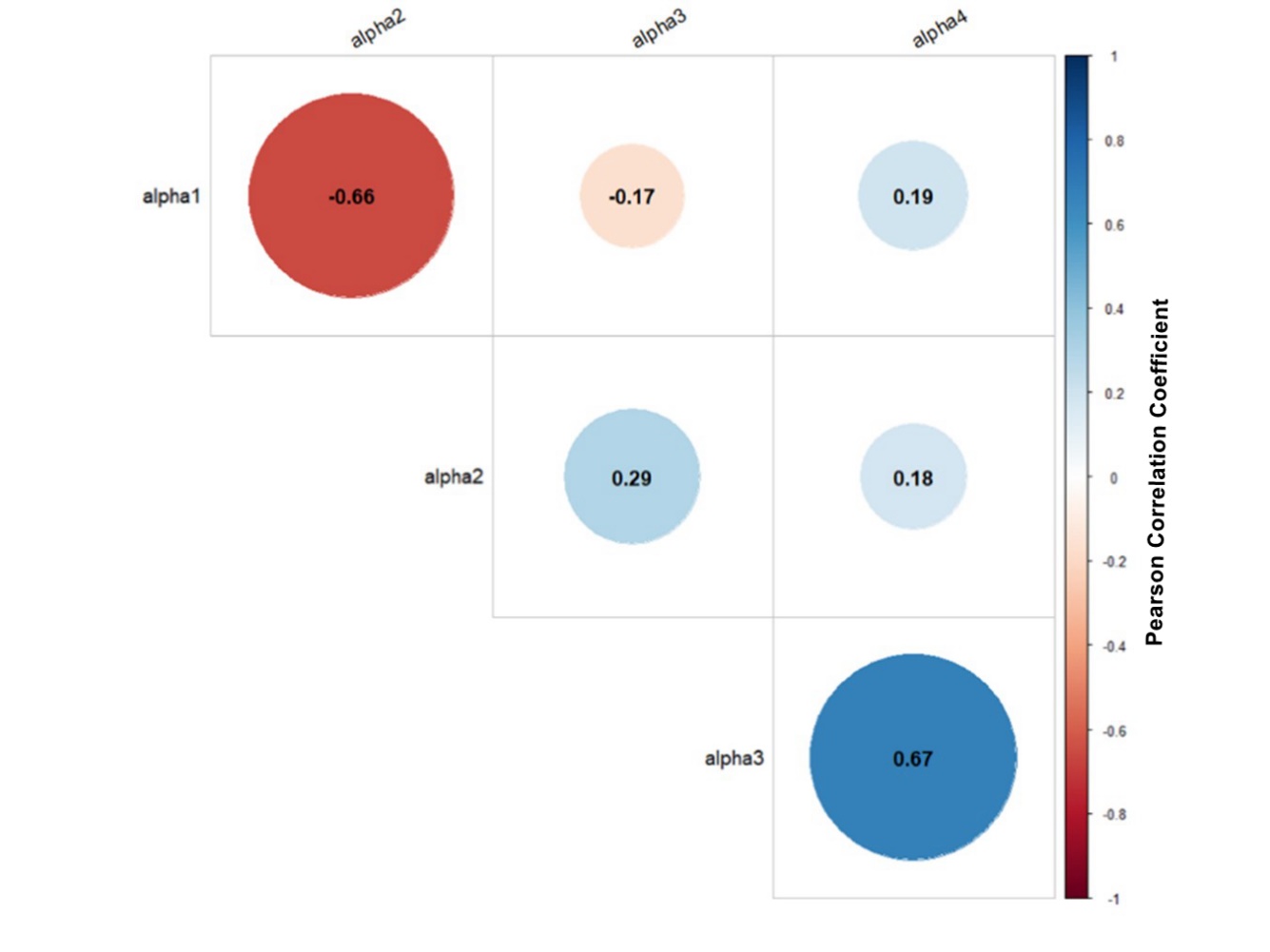


**Supplementary Figure 3 The correlation plot of individual random effects**

The random effects were extracted from Bayesian Bent-Line Regression model using the longitudinal PACC3 data. Numbers represent Pearson’s rho. PACC3 = Preclinical Alzheimer’s Cognitive Composite (comprising averaged z-scores for 3 tests: Auditory Verbal Learning Test (AVLT) Total Learning, Logical Memory Delayed Recall, and Digit Symbol Substitution). Abbreviations: alpha1: posterior median estimate intercepts at change points (CPs) from 5000*4 iterations; alpha2: median estimate slopes pre-CP; alpha3: median estimate slopes post-CP; and alpha4: median estimate person-level CPs for PACC3.


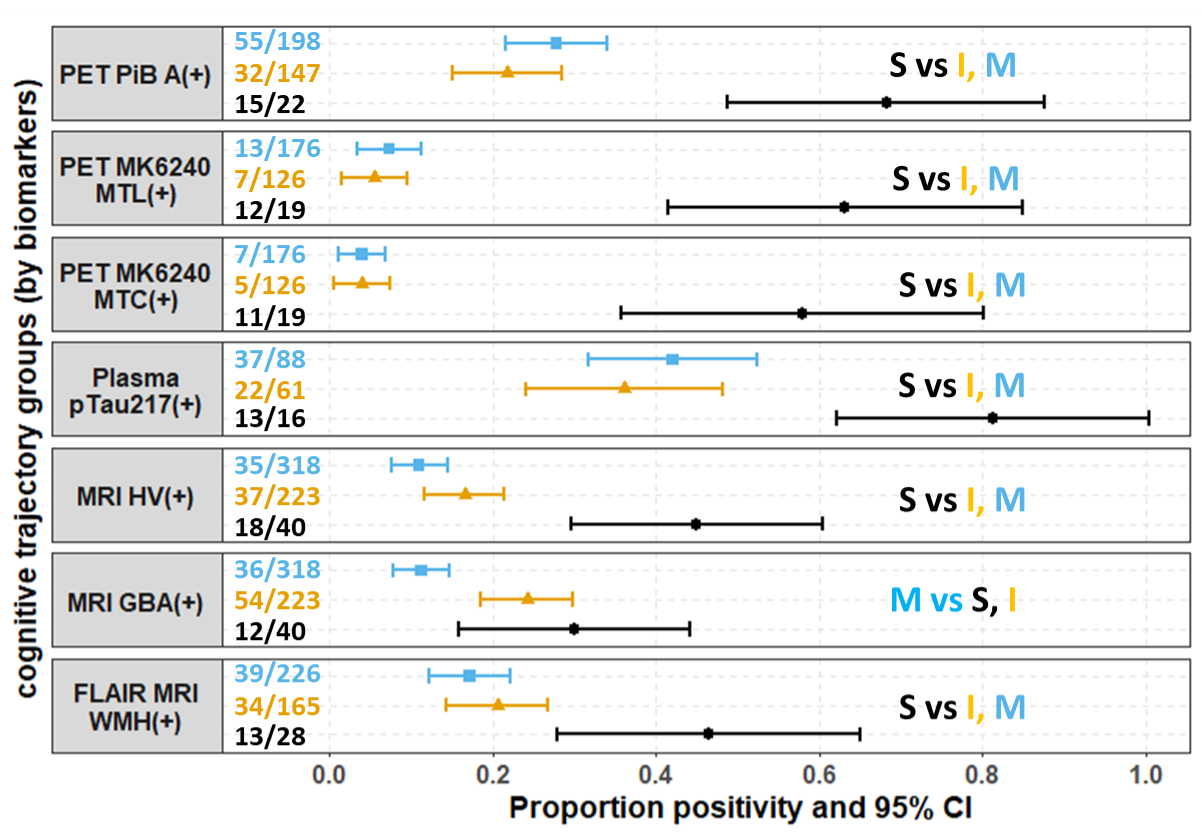


**Supplementary Figure 4 Comparison of most recent dementia biomarkers positivity (secondary) across** **cognitive trajectory groups**

The positivity was defined in methods (Secondary). The number in the figure is the n of biomarker positivity in each cognitive trajectory group. Kruskal‐Wallis tests were used. Post hoc pairwise group differences at Benjamini Hochberg adjusted P < 0.05 noted in the text. For example, “S vs I, M” indicates the Steep decline group differed from Intermediate and Minimal decline groups in separate pairwise comparisons. Compared to the primary method, more pTau217+, HV+, GBA+ and WMHV+ was identified using secondary method. Post hoc pairwise group differences were same to the primary method, except S differed I in WMHV positivity significantly. Abbreviations: S: Steep decline, I: Intermediate decline, M: Minimal decline, MTL: Medial temporal lobe composite (early tau stage composite; based on an average of the entorhinal cortex, hippocampus and amygdala) (Berron et al. 2021), MTC: Meta-temporal composite (mid-to-late tau stage composite, based on Mayo meta-temporal composite) (Jack, Wiste, et al. 2018), HV: Hippocampus Volume, GBA: Global brain atrophy, WMHV: White Matter Hyperintensities Volume.


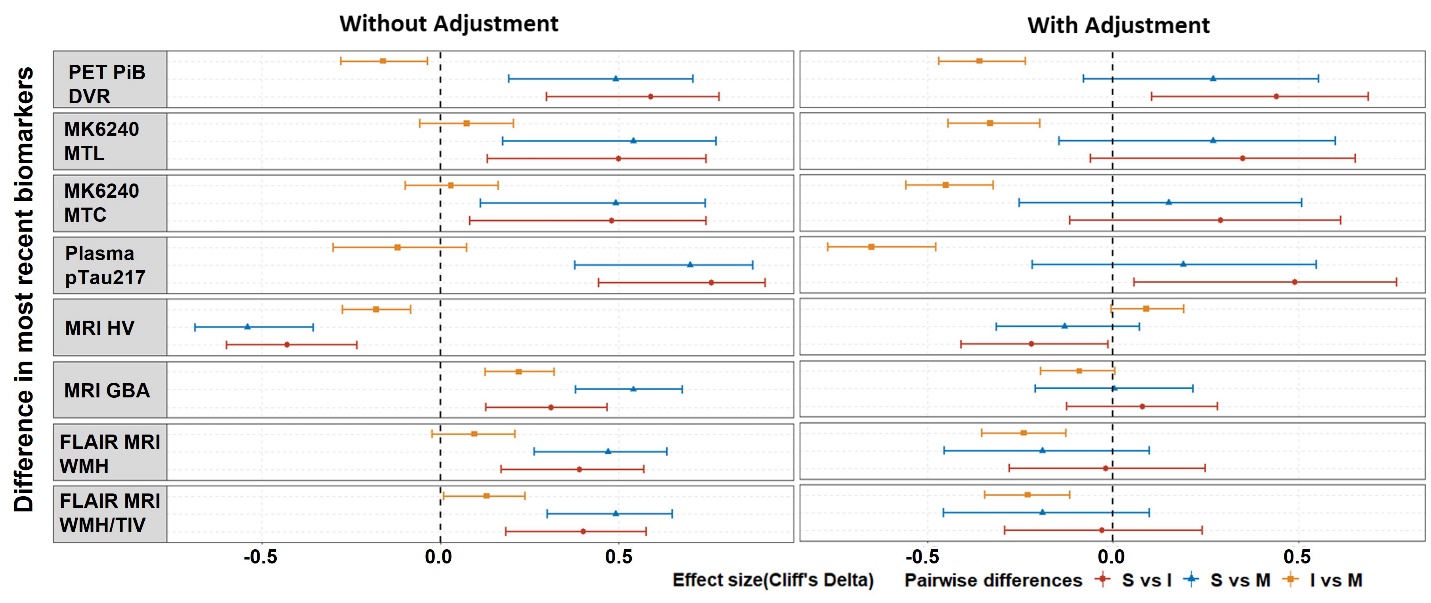


# Supplementary Figure 5 The effect size of difference in most recent PET PiB, MK-6240, Plasma and MRI outcomes among cognitive trajectory groups with and w/o adjustment

Age and Gender were adjusted in the adjusted model. The corresponding data are found in Fig. 2. The effect size is Cliff's delta. Abbreviations: S: Steep decline, I: Intermediate decline, M: Minimal decline, MTL: Medial temporal lobe composite (early tau stage composite; based on an average of the entorhinal cortex, hippocampus and amygdala) (Berron et al. 2021), MTC: Meta-temporal composite (mid-to-late tau stage composite, based on Mayo meta-temporal composite) (Jack, Wiste, et al. 2018), HV: Hippocampus Volume, GBA: Global brain atrophy, WMHV: White Matter Hyperintensities Volume.


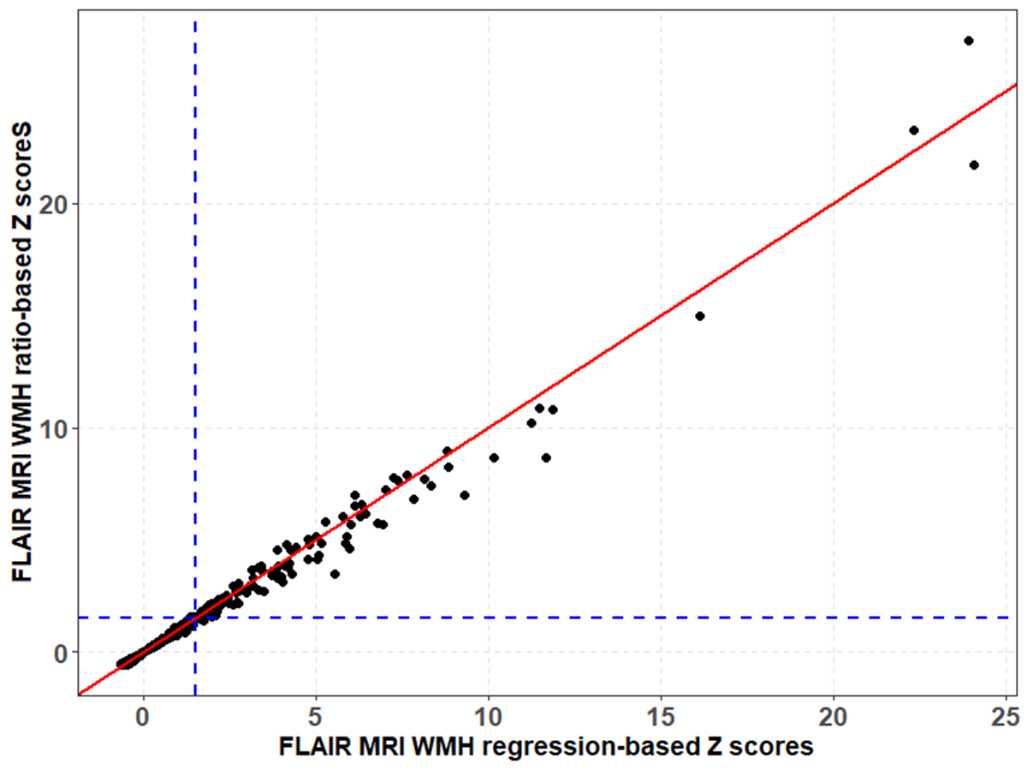


**Supplementary Figure 6 The scatter plot for WMH regression-based and ratio-based z-scores**

The red solid line is the diagonal line. Quadrant plots indicating the observed relationship between WMHV regression-based and ratio-based z-scores are shown in Figure with positivity thresholds (z = 1.5 approach) for these two z-scores indicated with blue dashed lines. To get WMHV regression-based Z scores: 1) using the CU, PET A- subset, we ran the regression equation: WMHV = intercept + beta*TICV and saved the beta’s and RMSE from this equation. Then, for everyone with WMHV, we used the regression equation to get predicted HV and calculate the z-score as: (Observed WMHV – Predicted WMHV)/(Root mean squared error from CU, A- subset regression model that adjusted for TICV). The equation to get WMHV ratio-based Z score was (Observed WMHV/TIV value – Mean value of CU, PET A-)/(Standard deviation of CU, PET A-). Abbreviations: WMH: White Matter Hyperintensities, WMHV: White Matter Hyperintensities Volume, TIV/TICV: total intracranial volume, CU: Cognitive unimpaired.


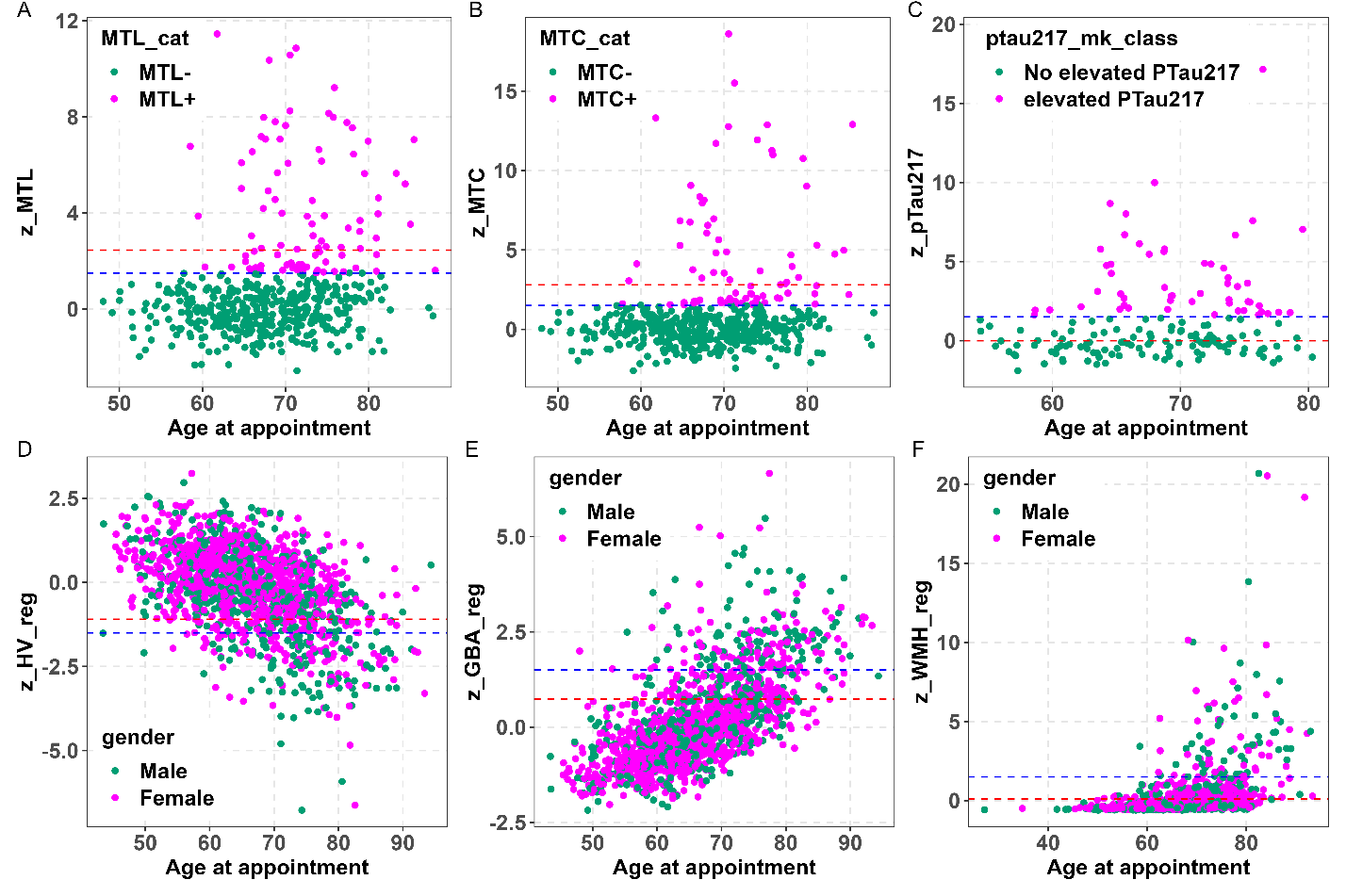


**Supplementary Figure 7 Biomarker thresholds across multiple biomarkers by the z = 1.5/-1.5 approach and by GMM**

The dots are colored by the classification using the threshold described by Cody et al (the 95%ile of the CU Aβ-negative (A-) participants) for MK6240 and the threshold that was defined by Jonaitis et al (ROC curve relative to MK tau+) for plasma pTau217. For HV, GBA, and WMHV, the dots are colored by Gender. The x-axis represents the age at appointment, and y-axis represents z-scores. Abbreviations: MTL: Medial temporal lobe composite (early tau stage composite; based on an average of the entorhinal cortex, hippocampus and amygdala) (Berron et al. 2021), MTC: Meta-temporal composite (mid-to-late tau stage composite, based on Mayo meta-temporal composite) (Jack, Wiste, et al. 2018), HV: Hippocampus Volume, GBA: Global brain atrophy, WMHV: White Matter Hyperintensities Volume, CU: Cognitive unimpaired.


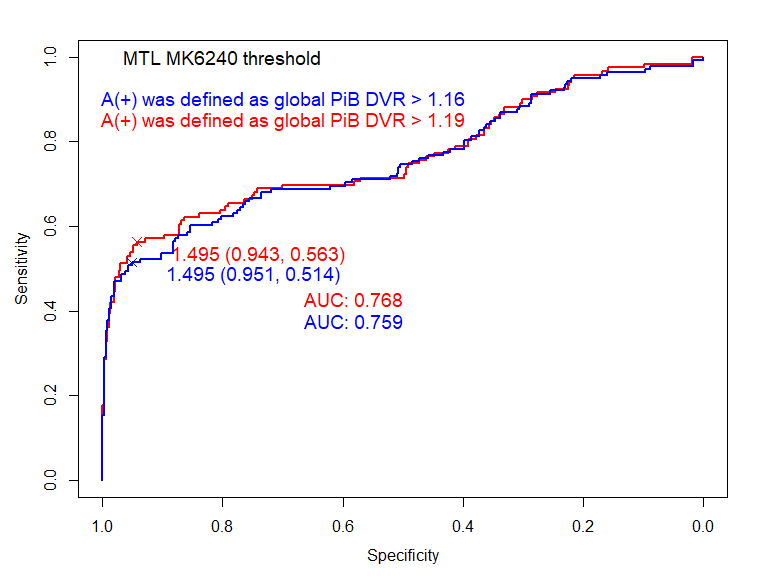


**Supplementary Figure 8 ROC curves of the MK6240 MTL z-scores relative to PET A-/+ status**

The corresponding raw score of MTL is 1.129 for the threshold recommended by the ROC curves relative to PiB PET A-/+ status. Two positivity thresholds were considered for PiB: global DVR > 1.19 (red) and global DVR > 1.16 (blue). The threshold (specificity, sensitivity) and AUC are presented in the figure. Sensitivity = TP / (TP+FN), Specificity = TN / (FP+TN). Abbreviations: DVR = distribution volume ratio, PiB = Pittsburgh compound B, pTau217 = phosphorylated tau 217, MTL: Medial temporal lobe composite, ROC: receiver–operator characteristic, AUC: Area Under the Curve, TP = True Positive, TN = True Negative, FP = False Positive, FN = False Negative.


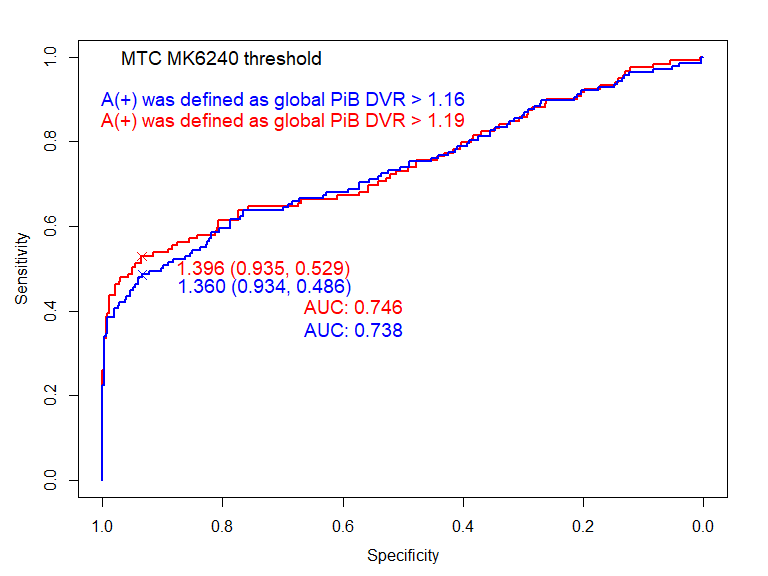


**Supplementary Figure 9 ROC curves of the MK6240 MTC z-scores relative to PET A-/+ status**

The corresponding raw score of MTC is 1.219, 1.224 for the threshold recommended by the ROC curves relative to PiB PET A-/+ status. Two positivity thresholds were considered for PiB: global DVR > 1.19 (red) and global DVR > 1.16 (blue). The threshold (specificity, sensitivity) and AUC are presented in the figure. Sensitivity = TP / (TP+FN), Specificity = TN / (FP+TN). Abbreviations: DVR = distribution volume ratio, PiB = Pittsburgh compound B, MTC: Meta-temporal composite, ROC: receiver–operator characteristic, AUC: Area Under the Curve, TP = True Positive, TN = True Negative, FP = False Positive, FN = False Negative.


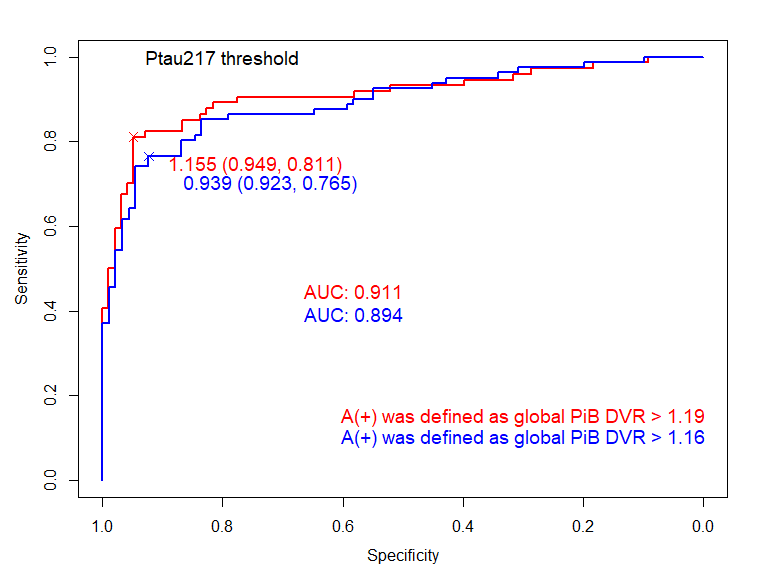


**Supplementary Figure 10 ROC curves of the Plasma pTau217 z-scores relative to PET A-/+ status**

The corresponding raw score of Plasma pTau217 is 0.323, 0.37 for the threshold recommended by the ROC curves relative to PiB PET A-/+ status. Two positivity thresholds were considered for PiB: global DVR > 1.19 (red) and global DVR > 1.16 (blue). The threshold (specificity, sensitivity) and AUC are presented in the figure. Sensitivity = TP / (TP+FN), Specificity = TN / (FP+TN). Abbreviations: DVR = distribution volume ratio, PiB = Pittsburgh compound B, pTau217 = phosphorylated tau 217, ROC: receiver–operator characteristic, AUC: Area Under the Curve, TP = True Positive, TN = True Negative, FP = False Positive, FN = False Negative.


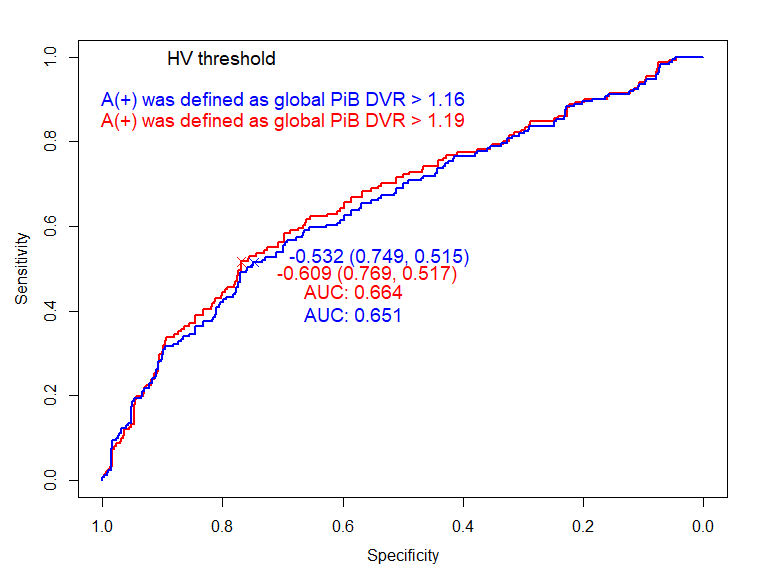


**Supplementary Figure 11 ROC curves of the MRI HV z-scores relative to PET A-/+ status**

The corresponding raw score of MRI HV is not available because we adjust TICV in the equation. Two positivity thresholds were considered for PiB: global DVR > 1.19 (red) and global DVR > 1.16 (blue). The threshold (specificity, sensitivity) and AUC are presented in the figure. Sensitivity = TP / (TP+FN), Specificity = TN / (FP+TN). Abbreviations: DVR = distribution volume ratio, PiB = Pittsburgh compound B, HV: Hippocampus Volume, ROC: receiver–operator characteristic, AUC: Area Under the Curve, TP = True Positive, TN = True Negative, FP = False Positive, FN = False Negative.


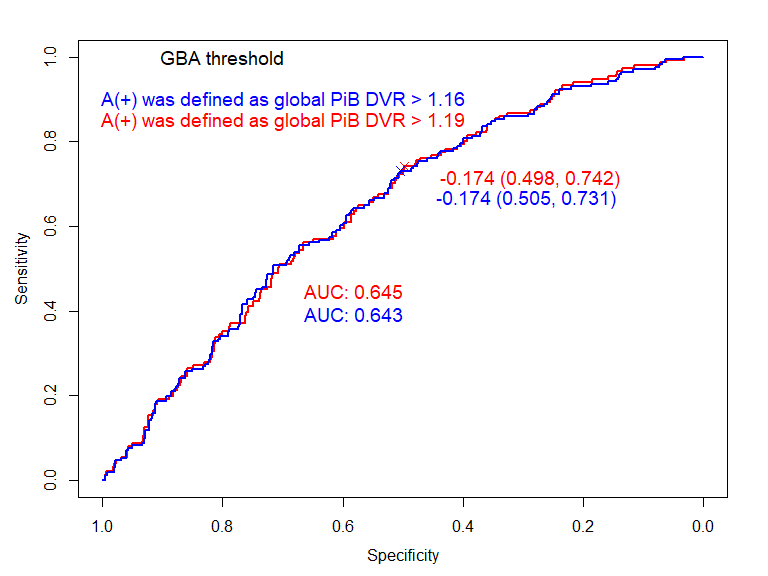


**Supplementary Figure 12 ROC curves of the MRI GBA z-scores relative to PET A-/+ status**

The corresponding raw score of MRI GBA is not available because we adjust TICV in the equation. Two positivity thresholds were considered for PiB: global DVR > 1.19 (red) and global DVR > 1.16 (blue). The threshold (specificity, sensitivity) and AUC are presented in the figure. Sensitivity = TP / (TP+FN), Specificity = TN / (FP+TN). Abbreviations: DVR = distribution volume ratio, PiB = Pittsburgh compound B, GBA: Global brain atrophy, ROC: receiver–operator characteristic, AUC: Area Under the Curve, TP = True Positive, TN = True Negative, FP = False Positive, FN = False Negative.


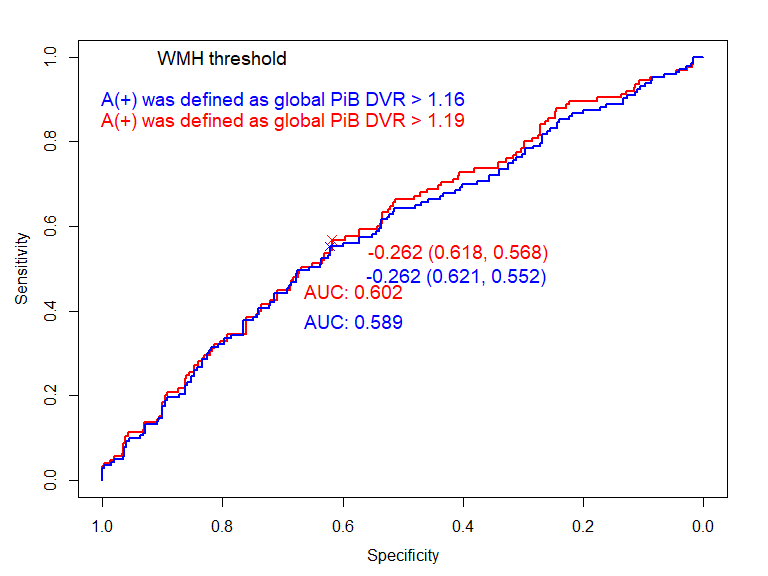


**Supplementary Figure 13 ROC curves of the FLAIR MRI WMH z-scores relative to PET A-/+ status**

The corresponding raw score of T2-FLAIR MRI WMHV is not available because we adjust TICV in the equation. Two positivity thresholds were considered for PiB: global DVR > 1.19 (red) and global DVR > 1.16 (blue). The threshold (specificity, sensitivity) and AUC are presented in the figure. Sensitivity = TP / (TP+FN), Specificity = TN / (FP+TN). Abbreviations: DVR = distribution volume ratio, PiB = Pittsburgh compound B, WMHV: White Matter Hyperintensities Volume, ROC: receiver–operator characteristic, AUC: Area Under the Curve, TP = True Positive, TN = True Negative, FP = False Positive, FN = False Negative.

**Supplementary Table 1. Characteristics of people who were included in the analyses and those who were excluded**

|  | Overall  (N = 1609) | Analyses data  (N = 1068) | Excluded data  (N = 541) | P-value^a^ |
| --- | --- | --- | --- | --- |
| Age at enrolment (years; mean (SD)) | 54.48 (6.8) | 54.14 (6.5) | 55.15 (7.4) | 0.005 |
| Age at first PACC3 (years; mean (SD))^b^ | 58.74 (6.6) | 58.40 (6.4) | 59.86 (7.2) | <0.001 |
| Female (%) | 1138 (70.7) | 738 (69.1) | 400 (73.9) | 0.05 |
| *APOE* e4 carriers (%) | 610 (39.7) | 407 (38.1) | 203 (43.3) | 0.064 |
| Family History of AD (%) | 1179 (73.4) | 795 (74.4) | 384 (71.2) | 0.191 |
| College degree (%) | 932 (58.0) | 677 (63.4) | 255 (47.4) | <0.001 |
| WRAT3 Reading (mean (SD)) | 104.57 (10.3) | 106.05 (9.0) | 101.61 (12.0) | <0.001 |
| AVLT Total^+^ | 50.46 (8.3) | 51.46 (7.9) | 48.51 (8.6) | <0.001 |
| Cognitive Status (at enrollment) (%) |  |  |  | <0.001 |
| CU-S | 1417 (88.1) | 965 (90.4) | 452 (83.5) |  |
| CU-D | 190 (11.8) | 102 (9.6) | 88 (16.3) |  |
| MCI | 2 (0.1) | 1 (0.1) | 1 (0.2) |  |

Note: Overall: Participants who were enrolled with at least a baseline visit at the time of these analyses; Analyses data: Participants who met enrolment criteria. Abbreviations: College degree, Education years>=16; WRAT3, wide range achievement test (third edition); PACC3, global cognitive composite 3; CU-S, cognitively unimpaired – stable; CU-D, cognitively unimpaired – declining; MCI, mild cognitive impairment.

^a^Statistical tests: chi‐square or Fisher's exact for categorical; analysis of variance (ANOVA) for continuous where M (SD) reported; Kruskal‐Wallis for continuous where median [Q1‐Q3] reported and Likert-scale items.

^b^Due to two tests needed to calculate PACC3 being added later in the study, the sample size for the first PACC3 age are N = 1391, 1068, and 323, respectively. AVLT Total was available since baseline and baseline mean(sd) are included here.

**Supplementary Table 2 Individual random effects, Overall and by Cognitive Trajectory Group**

|  | **Overall** | **Steep cognitive decline (N=77)** | **Intermediate cognitive decline  (N=446)** | **Minimal cognitive decline (N=545)** | **P-value**^a^ | **Difference pairs** |
| --- | --- | --- | --- | --- | --- | --- |
| **alpha1**  **(mean (SD))** | -0.01 (0.59) | -0.12 (0.40) | -0.51 (0.43) | 0.42 (0.35) | **<0.001** | All Pairs |
| **alpha2**  **(mean (SD))** | -0.01 (0.00) | -0.01 (0.00) | 0.00 (0.00) | -0.01 (0.00) | **<0.001** | I vs S, M |
| **alpha3**  **(median [IQR])** | -0.11 [-0.12,  -0.11] | -0.16 [-0.21, -0.14] | -0.11 [-0.11, -0.10] | -0.12 [-0.12, -0.11] | **<0.001** | All Pairs |
| **alpha4**  **(median [IQR])** | 75.75 [75.00, 76.77] | 65.72 [62.28, 67.81] | 75.54 [74.94, 76.80] | 76.12 [75.43, 76.86] | **<0.001** | All Pairs |

Note: alpha1 is the individual intercept denoting the expected value of the cognitive test score at the change point (CP), alpha2 is the individual linear slope before the CP (pre-change slope), alpha3 is the individual post-change slope, alpha4 is individual CP.

^a^Statistical tests: chi‐square or Fisher's exact for categorical; analysis of variance (ANOVA) for continuous where M (SD) reported; Kruskal‐Wallis for continuous where median [Q1‐Q3] reported and Likert-scale items. Post hoc pairwise group differences at Benjamini Hochberg adjusted P < 0.05 noted in right‐hand column. For example, “I vs S, M” indicates Intermediate decline group differed from Steep and Minimal decline groups in separate pairwise comparisons.

**Supplementary Table 3 Sample categorical Characteristics (Most recent), Overall and by Cognitive Trajectory Group**

|  | **Overall** | **Steep cognitive decline (N=77)** | **Intermediate cognitive decline  (N=446)** | **Minimal cognitive decline (N=545)** | **P-value**^a^ | **Difference pairs** |
| --- | --- | --- | --- | --- | --- | --- |
| **BMI (%)** |  |  |  |  | 0.362 |  |
| **Normal** | 282 ( 27.3) | 17 ( 23.9) | 110 ( 25.6) | 155 ( 29.0) |  |  |
| **Underweight** | 8 ( 0.8) | 0 ( 0.0) | 2 ( 0.5) | 6 ( 1.1) |  |  |
| **Overweight** | 345 (33.4) | 27 ( 38.0) | 140 ( 32.6) | 178 ( 33.3) |  |  |
| **Obese** | 399 (38.6) | 27 ( 38.0) | 177 ( 41.3) | 195 ( 36.5) |  |  |
| **WHR (%)** |  |  |  |  | <0.001 | M vs I, S |
| **Low** | 368 (35.8) | 24 ( 33.8) | 137 ( 32.1) | 207 ( 39.1) |  |  |
| **Moderate** | 292 (28.4) | 19 ( 26.8) | 123 ( 28.8) | 150 ( 28.3) |  |  |
| **High** | 368 (35.8) | 28 ( 39.4) | 167 ( 39.1) | 173 ( 32.6) |  |  |
| **Prescription (%)** |  |  |  |  | 0.004 | S vs I, M |
| **NP** | 737 (69.3) | 41 ( 53.9) | 305 ( 68.7) | 391 ( 71.9) |  |  |
| **PP** | 269 (25.3) | 27 ( 35.5) | 111 ( 25.0) | 131 ( 24.1) |  |  |
| **HP** | 58 ( 5.5) | 8 ( 10.5) | 28 ( 6.3) | 22 ( 4.0) |  |  |
| **Hypertension (%)** | 307 (36.0) | 25 ( 49.0) | 133 ( 36.8) | 149 ( 33.8) | 0.091 | M vs S |
| **Diabetes (%)** | 95 (11.1) | 12 ( 23.5) | 39 ( 10.8) | 44 ( 10.0) | 0.014 | S vs I, M |
| **HighCholesterol (%)** | 494 (57.9) | 38 ( 74.5) | 209 ( 57.9) | 247 ( 56.0) | 0.04 | S vs I, M |
| **Depression (%)** | 335 (39.3) | 27 ( 54.0) | 144 ( 40.1) | 164 ( 36.9) | 0.059 |  |
| **Stroke (%)** | 20 ( 2.3) | 3 ( 5.9) | 4 ( 1.1) | 13 ( 2.9) | 0.052 |  |
| **Heart (%)** | 257 (30.1) | 14 ( 27.5) | 108 ( 29.9) | 135 ( 30.6) | 0.891 |  |
| **SRH (%)** |  |  |  |  | 0.047 | M vs S |
| **Excellent** | 570 (53.6) | 33 ( 42.9) | 234 ( 52.7) | 303 ( 55.9) |  |  |
| **Good** | 419 (39.4) | 35 ( 45.5) | 176 ( 39.6) | 208 ( 38.4) |  |  |
| **Poor** | 74 ( 7.0) | 9 ( 11.7) | 34 ( 7.7) | 31 ( 5.7) |  |  |
| **SRCM (%)** |  |  |  |  | <0.001 | All Pairs |
| **No problem** | 696 (66.9) | 33 ( 45.8) | 270 ( 62.5) | 393 ( 73.2) |  |  |
| **Neutral Problem** | 226 (21.7) | 20 ( 27.8) | 107 ( 24.8) | 99 ( 18.4) |  |  |
| **Major problem** | 119 (11.4) | 19 ( 26.4) | 55 ( 12.7) | 45 ( 8.4) |  |  |
| **LIBRA Score Tertiles (%)** |  |  |  |  | 0.002 | M vs I |
| **Low** | 358 (33.5) | 24 ( 31.2) | 124 ( 27.8) | 210 ( 38.5) |  |  |
| **Moderate** | 359 (33.6) | 26 ( 33.8) | 157 ( 35.2) | 176 ( 32.3) |  |  |
| **High** | 351 (32.9) | 27 ( 35.1) | 165 ( 37.0) | 159 ( 29.2) |  |  |

Note: College =Y = Education years>=16. BMI = Body Mass Index. WHR = Waist to Hip Ratio. SRH = Self Rated Health. SRCM = Self-reported Cognitive Measures.

^a^Statistical tests: chi‐square or Fisher's exact for categorical; Kruskal‐Wallis for Likert-scale items. Post hoc pairwise group differences at Benjamini Hochberg adjusted P < 0.05 noted in right‐hand column. For example, “S vs I, M” indicates Steep decline group differed from Intermediate and Minimal decline groups in separate pairwise comparisons.

**Supplementary Table 4 Sample Continuous Characteristics (Baseline and Most recent), Overall and by Cognitive Trajectory Group**

|  | **Overall** | **Steep cognitive decline (N=77)** | **Intermediate decline  (N=446)** | **Minimal decline (N=545)** | **P-value**^a^ | **Difference pairs** |
| --- | --- | --- | --- | --- | --- | --- |
| **Baseline** |  |  |  |  |  |  |
| **BMI (median [IQR])** | 28.1 [24.6, 32.6] | 27.7 [25.0, 32.0] | 28.7 [25.4, 33.0] | 27.6 [24.2, 32.4] | 0.078 |  |
| **WHR (median [IQR])** | 0.86 [0.80, 0.95] | 0.85 [0.80, 0.94] | 0.89 [0.82, 0.97] | 0.84 [0.78, 0.92] | <0.001 | M vs I |
| **CES_D Score (median [IQR])** | 4.00 [2.00, 9.00] | 6.00 [3.00, 11.00] | 5.00 [2.00, 9.00] | 4.00 [2.00, 8.00] | 0.045 | M vs S |
| **Prescription (median [IQR])** | 2.00 [1.00, 5.00] | 4.00 [2.00, 6.00] | 3.00 [1.00, 5.00] | 2.00 [1.00, 4.00] | <0.001 | All Pairs |
| **Comorbidity (median [IQR])** | 3.00 [2.00, 5.00] | 4.00 [3.00, 6.00] | 4.00 [2.00, 6.00] | 3.00 [2.00, 5.00] | 0.059 |  |
| **LIBRA Score (median [IQR])** | 1.40 [0.00, 3.00] | 1.60 [0.00, 3.10] | 1.60 [0.10, 3.20] | 0.60 [0.00, 2.70] | <0.001 | M vs S, I |
| **Most recent** |  |  |  |  |  |  |
| **BMI (median [IQR])** | 28.2 [24.6, 33.0] | 27. 8 [25.0, 32.8] | 28.5 [24.9, 33.1] | 28.0 [24.3, 32.8] | 0.361 |  |
| **WHR (median [IQR])** | 0.87 [0.81, 0.95] | 0.88 [0.81, 0.95] | 0.90 [0.83, 0.98] | 0.85 [0.79, 0.92] | <0.001 | M vs I |
| **CES_D Score (median [IQR])** | 4.00 [1.00, 9.00] | 6.00 [3.00, 10.00] | 5.00 [1.00, 9.00] | 4.00 [1.00, 8.00] | 0.002 | M vs S |
| **Prescription (median [IQR])** | 3.00 [1.00, 5.00] | 4.00 [2.00, 6.25] | 3.00 [1.00, 5.00] | 3.00 [1.00, 5.00] | 0.001 | S vs I, M |
| **Comorbidity (median [IQR])** | 4.00 [2.00, 6.00] | 5.00 [3.00, 6.00] | 4.00 [2.00, 6.00] | 4.00 [2.00, 6.00] | 0.174 |  |
| **LIBRA Score (median [IQR])** | 1.30 [-0.50, 2.92] | 1.40 [-1.00, 3.20] | 1.60 [0.00, 3.20] | 0.80 [-0.60, 2.20] | 0.001 | M vs I |

^a^Statistical tests: chi‐square or Fisher's exact for categorical; analysis of variance (ANOVA) for continuous where M (SD) reported; Kruskal‐Wallis for continuous where median [Q1‐Q3] reported and Likert-scale items. Post hoc pairwise group differences at Benjamini Hochberg adjusted P < 0.05 noted in right‐hand column. For example, “S vs I, M” indicates Steep decline group differed from Intermediate and Minimal decline groups in separate pairwise comparisons.

**Supplementary Table 5** **The effect size of difference in most recent PET PiB, MK-6240, MRI and Plasma outcome among cognitive trajectory groups with and w/o adjustment**

|  | **Pairwise differences** | **Effect Size** | |
| --- | --- | --- | --- |
|  |  | **Without adjustment** | **With adjustment**^a^ |
| **PET PiB** |  |  |  |
| Global PiB DVR | S vs I | 0.59 | 0.44 |
|  | S vs M | 0.49 | 0.27 |
|  | I vs M | -0.16 | -0.36 |
| **PET MK-6240** |  |  |  |
| MTL Z score | S vs I | 0.50 | 0.35 |
|  | S vs M | 0.54 | 0.27 |
|  | I vs M | 0.07 | -0.33 |
| MTC Z score | S vs I | 0.48 | 0.29 |
|  | S vs M | 0.49 | 0.15 |
|  | I vs M | 0.03 | -0.45 |
| **Plasma** |  |  |  |
| pTau217 Z score | S vs I | 0.76 | 0.49 |
|  | S vs M | 0.70 | 0.19 |
|  | I vs M | -0.12 | -0.65 |
| **MRI** |  |  |  |
| HV Z score | S vs I | -0.43 | -0.22 |
|  | S vs M | -0.54 | -0.13 |
|  | I vs M | -0.18 | 0.09 |
| GBA Z score | S vs I | 0.31 | 0.08 |
|  | S vs M | 0.54 | 0.004 |
|  | I vs M | 0.22 | -0.09 |
| **FLAIR MRI** |  |  |  |
| WMH Z score | S vs I | 0.39 | -0.02 |
|  | S vs M | 0.47 | -0.19 |
|  | I vs M | 0.10 | -0.24 |

^a^Adjusted for age and sex/gender. The corresponding data are found in Figure 2. The effect size is Cliff's delta. Abbreviations: MTL: Medial temporal lobe composite (early tau stage composite; based on an average of the entorhinal cortex, hippocampus and amygdala) (Berron et al. 2021), MTC: Meta-temporal composite (mid-to-late tau stage composite, based on Mayo meta-temporal composite) (Jack, Wiste, et al. 2018), HV: Hippocampus Volume, GBA: Global brain atrophy, WMH: White Matter Hyperintensities Volume.

**Supplementary Table 6 Biomarker z-scores summary across multiple biomarkers.**

| Biomarkers | N | Equation | Parameter Values |
| --- | --- | --- | --- |
| PET MK6240 MTL (SUVR) | 500 | (Observed MTL value – Mean value of CU, PET A-)/(Standard deviation of CU, PET A-) | Mean value of CU, PET A- = 0.935;  Standard deviation of CU, PET A- = 0.13 |
| PET MK6240 MTC (SUVR) | 500 | (Observed MTC value – Mean value of CU, PET A-)/(Standard deviation of CU, PET A-) | Mean value of CU, PET A- = 1.057;  Standard deviation of CU, PET A- = 0.12 |
| Plasma pTau217 (pg/mL) | 172 | (Observed value – Mean value of CU, PET A-)/(Standard deviation of CU, PET A-) | Mean value of CU, PET A- = 0.252;  Standard deviation of CU, PET A- = 0.08 |
| MRI HV (mL) | 1509 | 1. HV = intercept + beta*TICV in CU, PET A- subset  2. Predicted HV = intercept + beta from equation 1*TICV in everyone with an HV  3. (Observed HV – Predicted HV)/(RMSE from equation 1) | intercept = 2844.324;  beta = 3475.348;  RMSE = 761.8575 |
| MRI GBA (L/L) | 1509 | 1. GBA = intercept + beta*TICV in CU, PET A- subset  2. Predicted GBA = intercept + beta from equation 1*TICV in everyone with an GBA  3. (Observed GBA – Predicted GBA)/(RMSE from equation 1) | intercept = 0.1311248;  beta = 0.1340080;  RMSE = 0.0831023 |
| FLAIR MRI WMH (L) | 1154 | 1. WMH = intercept + beta*TICV in CU, PET A- subset  2. Predicted WMH = intercept + beta from equation 1*TICV in everyone with an WMH  3. (Observed WMH – Predicted WMH)/(RMSE from equation 1) | intercept = 2.961687e-05;  beta = 1.093493e-03;  RMSE = 0.002863539 |

Note: GBA was calculated as the ratio of CSF volume to the sum of the grey matter and white matter tissue volumes.

Abbreviations: MTL: Medial temporal lobe composite (early tau stage composite; based on an average of the entorhinal cortex, hippocampus and amygdala) (Berron et al. 2021), MTC: Meta-temporal composite (mid-to-late tau stage composite, based on Mayo meta-temporal composite) (Jack, Wiste, et al. 2018), SUVR: standardized uptake volume ratio, HV: Hippocampus Volume, GBA: Global brain atrophy, WMH: White Matter Hyperintensities Volume, TICV: Total intracranial volume, RMSE: Root mean squared error.

**Supplementary Table 7 Biomarker positivity proportions summary across multiple biomarkers.**

|  | Using Others' Cut-offs (published, or under review) | | | z = 1.5/-1.5 approach | | | GMM (> 50% probability) | | | ROC curves  Global PiB DVR=1.16 as cut off | | | |
| --- | --- | --- | --- | --- | --- | --- | --- | --- | --- | --- | --- | --- | --- |
|  | raw value cut | z-value cut | n(%) abnor-mal | raw value cut | z-value cut | n(%) abnor-mal | raw value cut | z-value cut | n(%) abnor-mal | raw value cut | z-value cut | n(%) abnor-mal | AUC (CI) |
| PET MK6240 MTL | 1.13^a^ | 1.5 | 90 (18.0) | 1.13 | 1.5 | 90 (18.0) | 1.25 | 2.45 | 56 (11.2) | 1.129 | 1.495 | 91 (18.2) | 0.759 (0.514, 0.951) |
| PET MK6240 MTC | 1.24 | 1.53 | 76 (15.2) | 1.237 | 1.5 | 79 (15.8) | 1.39 | 2.8 | 42 (8.4) | 1.219 | 1.36 | 91 (18.2) | 0.738 (0.486, 0.934) |
| Plasma pTau217 | 0.37^b^ | 1.5 | 57 (33.1) | 0.37 | 1.5 | 57 (33.1) | 0.252 | 0 | 74 (43.0) | 0.323 | 0.939 | 69 (40.1) | 0.894 (0.765, 0.923) |
| MRI HV | 4.9^c^ | | 425 (28.2) |  | -1.5 | 209 (13.9) |  | -1.1 | 308 (20.4) |  | -0.532 | 522 (34.6) | 0.651 (0.515, 0.749) |
| MRI GBA | 0.39 |  | 562 (37.2) |  | 1.5 | 203 (13.5) |  | 0.74 | 380 (25.2) |  | -0.174 | 683 (45.3) | 0.643 (0.505, 0.731) |
| FLAIR MRI WMH | |  |  |  | 1.5 | 103(8.9) |  | 0.11 | 286(24.8) |  | -0.262 | 459 (42.9) | 0.589 (0.552, 0.621) |

Note: ^a^Per work by Cody et al (*In review*), MTL = 1.13

^b^Per work by Jonaitis et al (ROC curve relative to MK tau+), pTau217 = 0.37

^c^Per work by Allison et al (Allison et al., 2019), HV/TICV=4.9

**Supplementary references**

Allison, S. L., Koscik, R. L., Cary, R. P., Jonaitis, E. M., Rowley, H. A., Chin, N. A., Zetterberg, H., Blennow, K., Carlsson, C. M., Asthana, S., Bendlin, B. B., & Johnson, S. C. (2019). Comparison of different MRI-based morphometric estimates for defining neurodegeneration across the Alzheimer’s disease continuum. *NeuroImage : Clinical*, *23*, 101895. https://doi.org/10.1016/j.nicl.2019.101895

Betthauser, T. J., Bilgel, M., Koscik, R. L., Jedynak, B. M., An, Y., Kellett, K. A., Moghekar, A., Jonaitis, E. M., Stone, C. K., Engelman, C. D., Asthana, S., Christian, B. T., Wong, D. F., Albert, M., Resnick, S. M., Johnson, S. C., & Alzheimer’s Disease Neuroimaging Initiative. (2022). Multi-method investigation of factors influencing amyloid onset and impairment in three cohorts. *Brain: A Journal of Neurology*, awac213. https://doi.org/10.1093/brain/awac213

Du, L., Koscik, R. L., Betthauser, T. J., Johnson, S. C., Larget, B., & Chappell, R. (2022). *BAyesian Bent-Line Regression model for longitudinal data with an application to the study of cognitive performance trajectories in Wisconsin Registry for Alzheimer’s Prevention*. https://doi.org/10.48550/arXiv.2211.09915

Farrell, M. E., Jiang, S., Schultz, A. P., Properzi, M. J., Price, J. C., Becker, J. A., Jacobs, H. I. L., Hanseeuw, B. J., Rentz, D. M., Villemagne, V. L., Papp, K. V., Mormino, E. C., Betensky, R. A., Johnson, K. A., Sperling, R. A., & Buckley, R. F. (2021). Defining the Lowest Threshold for Amyloid-PET to Predict Future Cognitive Decline and Amyloid Accumulation. *Neurology*, *96*(4), e619–e631. https://doi.org/10.1212/WNL.0000000000011214

Jonaitis, E. M., Janelidze, S., Cody, K. A., Langhough, R., Du, L., Chin, N. A., Mattsson-Carlgren, N., Hogan, K. J., Christian, B. T., Betthauser, T. J., Hansson, O., & Johnson, S. C. (2023). Plasma phosphorylated tau-217 in preclinical Alzheimer’s disease. *Brain Communications*, fcad057. https://doi.org/10.1093/braincomms/fcad057

Malone, I. B., Leung, K. K., Clegg, S., Barnes, J., Whitwell, J. L., Ashburner, J., Fox, N. C., & Ridgway, G. R. (2015). Accurate automatic estimation of total intracranial volume: A nuisance variable with less nuisance. *NeuroImage*, *104*, 366–372. https://doi.org/10.1016/j.neuroimage.2014.09.034

Racine, A. M., Clark, L. R., Berman, S. E., Koscik, R. L., Mueller, K. D., Norton, D., Nicholas, C. R., Blennow, K., Zetterberg, H., Jedynak, B., Bilgel, M., Carlsson, C. M., Christian, B. T., Asthana, S., & Johnson, S. C. (2016). Associations between performance on an abbreviated CogState battery, other measures of cognitive function, and biomarkers in people at risk for Alzheimer’s disease. *Journal of Alzheimer’s Disease : JAD*, *54*(4), 1395–1408. https://doi.org/10.3233/JAD-160528
